# New Terpenoids from *Potentilla freyniana* Bornm. and Their Cytotoxic Activities

**DOI:** 10.3390/molecules27123665

**Published:** 2022-06-07

**Authors:** Jia Wu, Zai-Qi Zhang, Xu-Dong Zhou, Qing-Ying Yao, Zhu-Liang Chen, Ling-Ling Chu, Huang-He Yu, Yu-Pei Yang, Bin Li, Wei Wang

**Affiliations:** 1 TCM and Ethnomedicine Innovation and Development International Laboratory, School of Pharmacy, Hunan University of Chinese Medicine, Changsha 410208, China; a17607310485@163.com (J.W.); xudongzhou999@163.com (X.-D.Z.); yaoqyyqy@163.com (Q.-Y.Y.); a844135048@163.com (Z.-L.C.); 13101112670@163.com (L.-L.C.); yhh@hnucm.edu.cn (H.-H.Y.); yangyupei24@163.com (Y.-P.Y.); 2 Hunan Provincial Key Laboratory of Dong Medicine, Hunan University of Medicine, Huaihua 418000, China; qizaizhang@126.com

**Keywords:** *Potentilla freyniana* Bornm., A-ring contracted triterpenoids, *ent*-kaurane diterpenoids, cytotoxic activities, Dong ethnomedicine

## Abstract

Two new A-ring contracted triterpenoids, madengaisu A and madengaisu B, and one undescribed *ent*-kaurane diterpenoid, madengaisu C, along with 20 known compounds were isolated from the roots of *Potentilla freyniana* Bornm. The structures were elucidated using extensive spectroscopic techniques, including 1D and 2D-NMR, HR-ESI-MS, ECD spectra, IR, and UV analysis. Moreover, all isolated constituents were evaluated for their anti-proliferative activity against RA-FLS cells and cytotoxic activities against the human cancer cell lines Hep-G2, HCT-116, BGC-823, and MCF-7. Ursolic acid and pomolic acid displayed moderate inhibitory activity in RA-FLS cells with IC_50_ values of 24.63 ± 1.96 and 25.12 ± 1.97 μM, respectively. Hyptadienic acid and 2*α*,3*β*-dihydroxyolean-12-en-28-oic acid 28-*O*-*β*-d-glucopyranoside exhibited good cytotoxicity against Hep-G2 cells with IC_50_ values of 25.16 ± 2.55 and 17.66 ± 1.82 μM, respectively. In addition, 2*α*,3*β*-dihydroxyolean-13(18)-en-28-oic acid and alphitolic acid were observed to inhibit HCT-116 cells (13.25 ± 1.65 and 21.62 ± 0.33 μM, respectively), while madengaisu B and 2*α*,3*β*-dihydroxyolean-13(18)-en-28-oic acid showed cytotoxic activities against BGC-823 cells with IC_50_ values of 24.76 ± 0.94 and 26.83 ± 2.52 μM, respectively, which demonstrated that triterpenes from *P. freyniana* may serve as therapeutic agents for RA and cancer treatment.

## 1. Introduction

*Potentilla* L. is a major genus belonging to the family Rosaceae with more than 200 species in the world, and many of them are widely used as ethnomedicine. Previous phytochemical research has shown that the genus *Potentilla* L. contains amounts of bioactive compounds, such as triterpenoids, flavonoids, and tannins, etc. Pharmacological studies also reported that *Potentilla* species possess significant hypoglycemic, anti-inflammatory, anti-tumor, antioxidative, and antibacterial activities [1].

*Potentilla freyniana* Bornm. is a perennial herb and is widely distributed in China, Russia, Japan, and Korea. It is also called “Ma Deng Ai” or “San Zhang ye” in the Dong ethnomedicine of China. The commonly used medicinal parts of the herb include the roots and whole grass, which have the effect of clearing heat and detoxifying, dispersing blood stasis, and stopping bleeding for the treatment of trauma bleeding, enteritis dysentery, stomatitis, and other diseases [2]. Previous phytochemical studies on *P. freyniana* afforded 29 compounds, mainly including flavonoids and triterpenoids, but there have been few investigations on biological activities [3].

**Figure 1:**
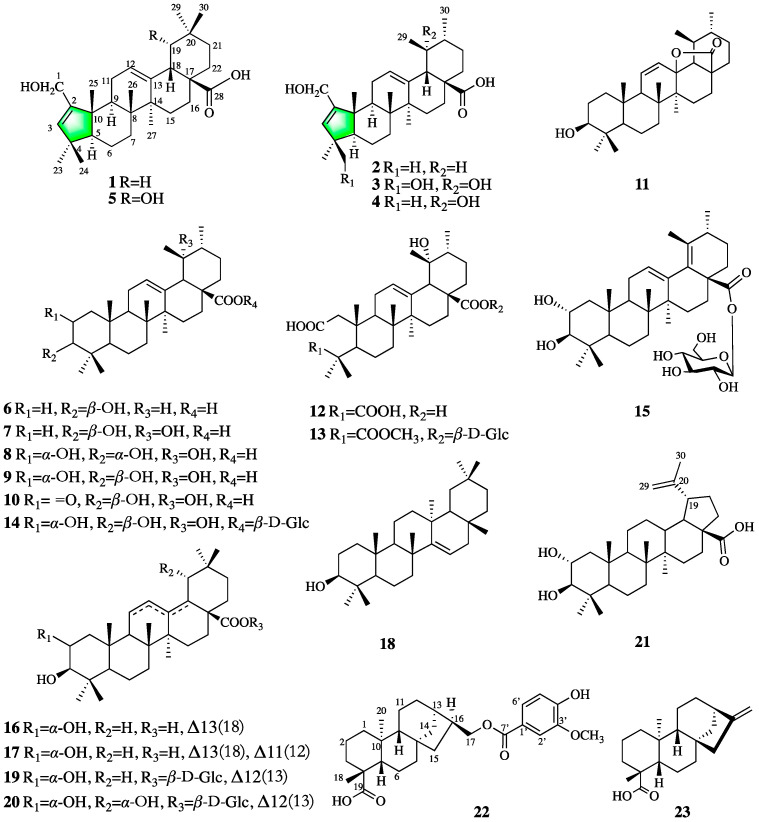
Chemical structures of compounds **1–23**.

To further investigate the pharmacologically active constituents of *P. freyniana*, a phytochemical study on the roots of *P. freyniana* was conducted, which led to the isolation of two undescribed A-ring contracted triterpenoids (**1** and **2**) and one new *ent*-kaurane diterpenoid (**22**) along with 20 known compounds (Figure 1) from the CH_2_Cl_2_-soluble layer. All isolates (**1–23**) were evaluated for their cytotoxic activities against RA-FLS cells and human cancer cell lines, including HepG2 (human liver carcinoma), HCT-116 (human colorectal carcinoma), BGC-823 (human gastric carcinoma), and MCF-7 (human breast carcinoma), to explore biologically significant metabolites. Herein we deal with the isolation and structure elucidation of the new compounds (**1–2**, **22**) as well as their anti-proliferative activity against RA-FLS cells and cytotoxic activities against the human cancer cell lines Hep-G2, HCT-116, BGC-823, and MCF-7.

## 2. Results

### 2.1. Structural Elucidation

**Figure 2:**
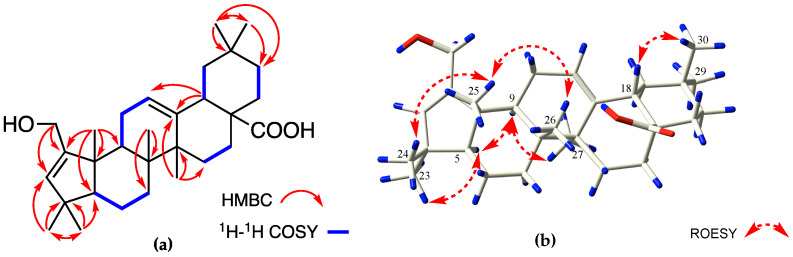
(**a**) Key ^1^H-^1^H COSY and HMBC correlations of **1**. (**b**) Selected ROESY correlations of **1**.

Compound **1** was obtained as a white and amorphous powder, and the molecular formula was determined to be C_30_H_46_O_3_ by HR-ESI-MS (*m*/*z* 477.3342 [M + Na]^+^, calculated for C_30_H_46_NaO_3,_ 477.3345), indicating eight degrees of unsaturation (Figure S3). The IR spectrum showed characteristic absorptions (Figure S2) of hydroxyl (3325 cm^−1^), methyl (2928, 2865, 1462, and 1381 cm^−1^), double bond (1605 cm^−1^), and ester carbonyl groups (1694 cm^−1^). In the ^1^H-NMR spectrum, seven tertiary methyls at *δ*_H_ 0.87, 0.91, 0.95, 0.96, 1.04, 1.16, and 1.20 (each 3H, s); an oxygen-bearing methylene group at *δ*_H_ 4.16 (dd, *J* = 14.5, 1.8 Hz, 1H) and 4.09 (dd, *J* = 14.5, 1.7 Hz, 1H); and two olefinic protons at *δ*_H_ 5.40 (s, 1H) and 5.25 (t, *J* = 3.5 Hz, 1H) were observed (Table 1). The ^13^C-NMR data with the aid of the DEPT-135° spectrum revealed 30 carbons attributed to seven methyls, nine methylenes, five methines (two olefinic carbons at *δ*_C_ 135.4 and 123.6), and nine quaternary carbons (two olefinic carbons at *δ*_C_ 156.0 and 145.7, and one carbonyl carbon at *δ*_C_ 181.9). Additionally, the degree of unsaturation of **1** and the presence of eight terminal carbons indicated a pentacyclic system containing two double bonds and one carboxyl group with a contracted A-ring rather than a *seco* structure. The 1D-NMR signals of **1** (Table 1) were similar to those of A-ring contracted oleanane sculponeatic acid (**5**), except that there was no resonance corresponding to a hydroxyl group on C-19 (*δ*_C_: 47.0) in **1** [4]. The ^1^H-^1^H COSY spectrum showed the presence of four independent spin systems (H-5/H_2–_6/H_2_-7, H-9/H_2_-11/H-12, H_2_-15/H_2_-16, and H_2_-21/H_2_-22), and interpretation of the HMBC of H_2_-1 with C-2 and C-3; H-12 with C-11 and C-18; H_3_-23 with C-24, C-3, C-4, and C-5; H_3_-24 with C-23, C-4, and C-5; H_3_-25 with C-2, C-5, C-9, and C-10; H_3_-26 with C-7 and C-8; H_3_-27 with C-13, C-14, and C-15; and H_3_-29 with C-19, C-20, C-21, and C-30 finally confirmed the planar structure of **1** (Figure 2a). To the best of our knowledge, this type of triterpenoid was rarely reported before.

**Figure 3:**
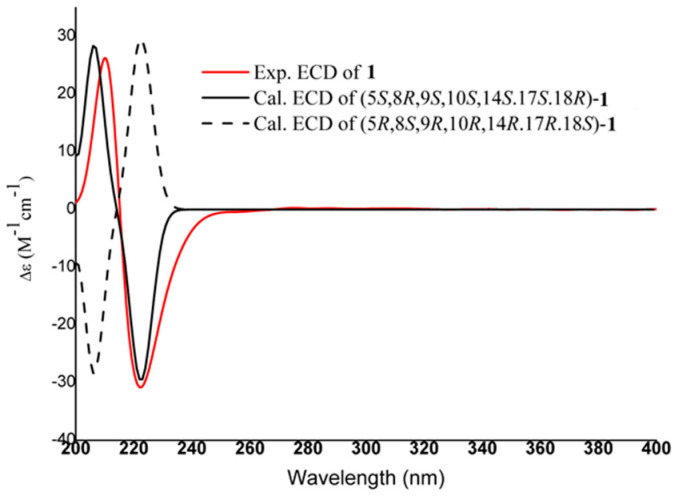
Experimental and calculated ECD spectra of **1**.

The relative configuration of **1** was ascertained through interpretation of its ROESY spectrum (Figure 2b). Based on the NOE correlations of H-5 with H_3_-9 and H_3_-23 and H-9 with H_3_-27, these protons were classified as *α*-oriented; the correlations of H_3_-25 with H_3_-24 and H_3_-26 and H-18 with H_3_-30 revealed their *β*-orientation. To further elucidate its absolute configuration, the electronic circular dichroism (ECD) spectrum of **1** was recorded in MeOH, and it showed a good agreement with the calculated ECD data of the (5*S*, 8*R*, 9*S*, 10*S*, 14*S*, 17*S*, 18*R*) model (Figure 3), which supported that the absolute configuration of **1** should be identical with 5*S*, 8*R*, 9*S*, 10*S*, 14*S*, 17*S*, 18*R*. Hence, the structure of **1** was established as 2-hydroxymethyl-1-norolean-2,12-dien-29-oic acid and it was named madengaisu A.

**Figure 4:**
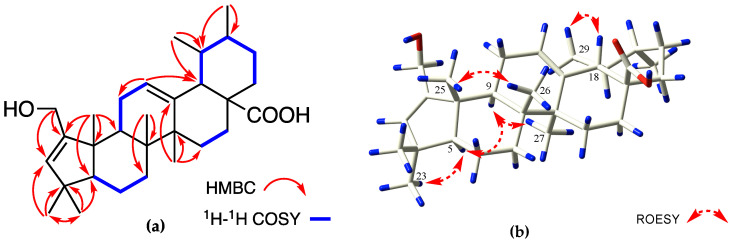
(**a**) Key ^1^H-^1^H COSY and HMBC correlations of **2**. (**b**) Selected ROESY correlations of **2**.

**Figure 5:**
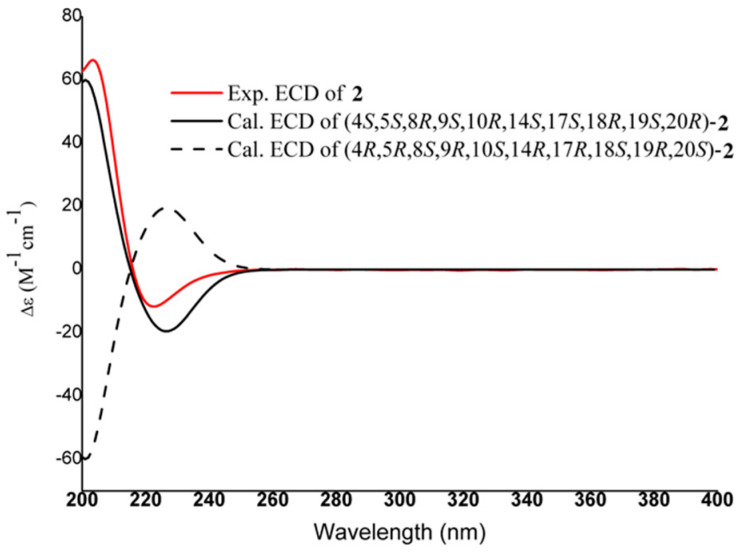
Experimental and calculated ECD spectra of **2**.

Compound **2** appeared as a white and amorphous powder. The molecular formula C_30_H_46_O_3_ and eight degrees of unsaturation are the same as those of **1**, based on HR-ESI-MS (*m*/*z* 477.3342 [M + Na]^+^, calculated for C_30_H_46_NaO_3_, 477.3361) and ^13^C NMR data (Figure S12). The NMR data (Table 1) of **2** were almost identical with those of compound **1**, except that there was a ursane-type skeleton instead of oleanane; hence, the presence of five tertiary methyls at *δ*_H_ 0.90, 0.95, 1.03, 1.15, and 1.16 (each 3H, s) and two methyl doublets at *δ*_H_ 0.96 (d, *J* = 4.8 Hz, 3H) and 0.90 (d, *J* = 6.6 Hz, 3H) in the ^1^H-NMR spectrum was inferred. Additionally, a H-18/H-19/H-20/H_2_-21/H_2_-22 long fragment of the ^1^H-^1^H COSY spectrum and HMBC correlations also supported the planar structure of **2** (Figure 4). Its stereochemistry is similar to that of **1**, and the *β*-orientation of H_3_-29 was deduced from the ROESY cross-peaks between H-18 and H_3_-29 (Figure 4). In the experimental ECD data, **2** showed a positive cotton effect at 203.4 nm and a negative cotton effect at 222.6 nm, which was consistent with the calculated ECD data of the (4*S*, 5*S*, 8*R*, 9*S*, 10*R*, 14*S*, 17*S*, 18*R*, 19*S*, 20*R*) model (Figure 5). Thus, the structure of **2** was established as 2-hydroxymethyl-1-norursa-2,12-dien-29-oic acid and it was named madengaisu B accordingly.

**Figure 6:**
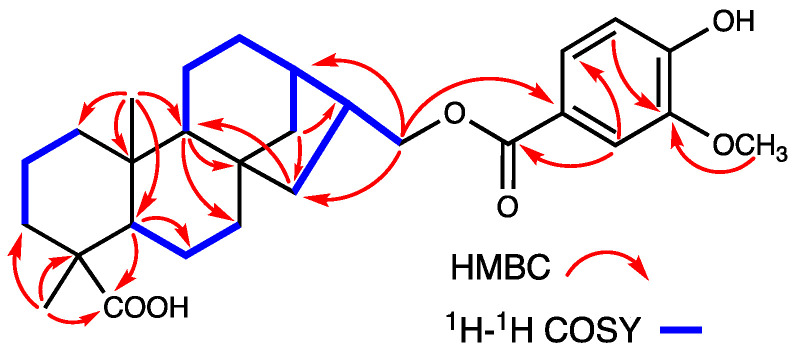
Key 2D-NMR correlations of compound **22**.

**Figure 7:**
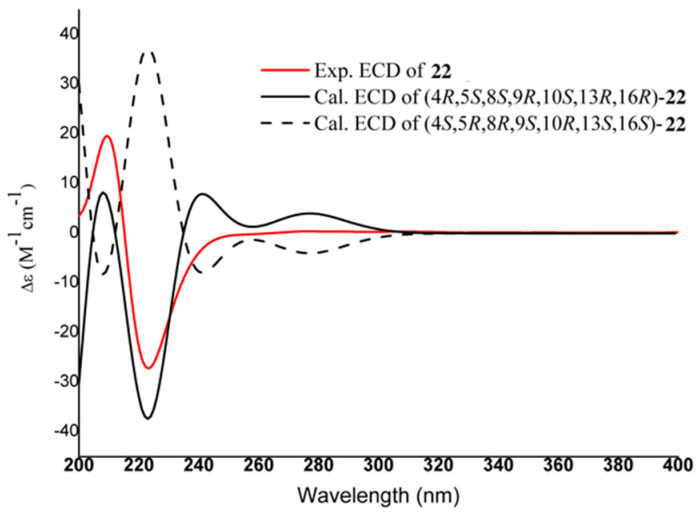
Experimental and calculated ECD spectra of compound **22**.

Compound **22** was purified as a white amorphous powder. It gave the molecular formula C_28_H_38_O_6_, with 10 degrees of unsaturation, based on HR-ESI-MS (*m*/*z* 493.2556 [M + Na]^+^, calculated for C_28_H_38_NaO_6_, 493.2566). The ^13^C-NMR and DEPT 135° spectra of **22** displayed 28 carbon resonances, including three methyls (one methoxyl at *δ*_C_ 56.4), ten methylenes, seven methines (three olefinic carbons at *δ*_C_ 113.5, 116.0, and 125.0), and eight quaternary carbons (three olefinic carbons at *δ*_C_ 122.8, 148.8, and 152.9 and two carbonyl carbons at *δ*_C_ 168.4 and 182.4). In addition, two methyl singlets at *δ*_H_ 0.99 and 1.19 (each 3H, s) were apparent in the ^1^H-NMR spectrum. The above 1D-NMR signals (Table 2) were similar to those of an *ent*-kaurane type diterpenoid, (-)-17-hydroxy-16*α*-*ent*-kauran-19-oic acid [5]. However, one methoxyl singlet at *δ*_H_ 3.90 (3H, s) and three aromatic protons at *δ*_H_ 6.85 (1H, d, *J* = 8.8 Hz), 7.54 (1H, d, *J* = 2.0 Hz), and 7.55 (1H, dd, *J* = 8.7, 2.0 Hz) attributed to a vanilloyl unit were also observed, suggesting that **22** contains a 1,3,4-trisubstituted phenyl ring. Furthermore, the spin system of H_2_-15/H-16/H-17 deduced from the ^1^H-^1^H COSY cross-peaks along with the HMBC correlations from H-2′, H_2_-17 to C-7′, and H_2_-17 to C-13 and C-15 illustrated that the hydroxyl at C-17 of the *ent*-kaurane skeleton was esterified by a vanilloyl group of **22**. Thus, the planar structure of **22** is shown in Figure 6. The ECD spectrum (Figure 7) of **22** was also recorded using the TDDFT method to give a result coincident with the experimental spectrum, possessing the absolute configuration of 4*R*, 5*S*, 8*S*, 9*R*, 10*S*, 13*R*, 16*R*. Therefore, the structure of **22** was identified as 17-(4′-hydroxy-3′-methoxybenzoate)-kaur-16-en-19-oic acid, and it was given the name madengaisu C.

The structures of the following twenty known compounds were identified by comparison of spectroscopic data with the reported literature and ESI-MS analyses: rosamultic acid (**3**) [6], hyptadienic acid (**4**) [4], sculponeatic acid (**5**) [4], ursolic acid (**6**) [7], pomolic acid (**7**) [8], euscaphic acid (**8**) [9], tormentic acid (**9**) [10], 2-oxo-pomolic acid (**10**) [11], 3-Hydroxy-13,28-epoxyurs-11-en-28-one (**11**) [12], cecropiacic acid (**12**) [13], potentillanoside E (**13**) [14], rosamultin (**14**) [15], rubuside A (**15**) [16], 2*α*,3*β*-dihydroxyolean-13(18)-en-28-oic acid (**16**) [17], camaldulenic acid (**17**) [18], taraxerol (**18**) [19], 2*α*,3*β*-dihydroxyolean-12-en-28-oic acid 28-*O*-*β*-d-glucopyranoside (**19**) [20], arjunetin (**20**) [21], alphitolic acid (**21**) [22], and (-)-kaur-16-en-19-oic acid (**23**) [23].

**Figure 8:**
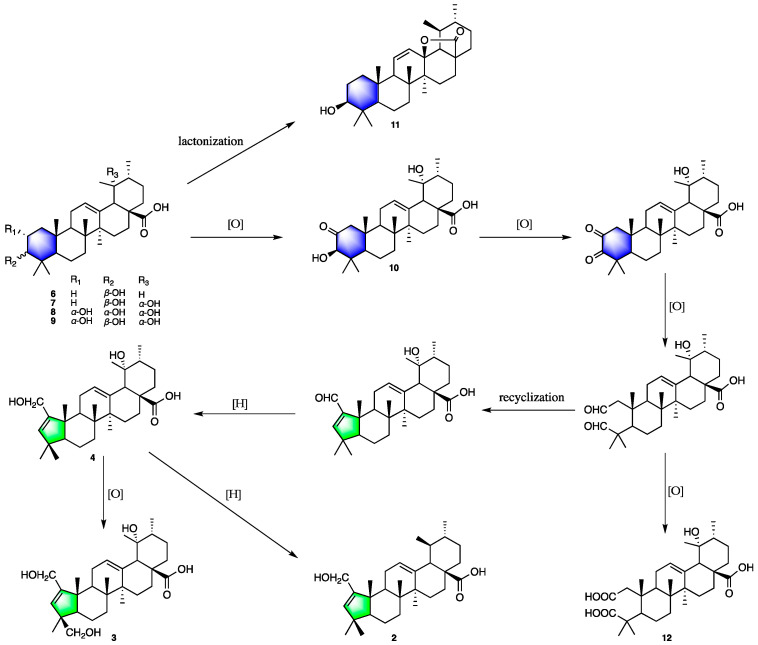
Plausible biosynthetic pathway for **2**–**4** and **6**–**12**.

Previous reports have shown that an A-ring contracted skeleton was found in ursane-, oleanane-, and dammarane-type triterpenes. In general, the *O*-containing groups at the C-2 or C-3 position of the six-membered A-ring go through oxidation reactions, which leads to selective C(2)-C(3) bond cleavage and further recyclization to form an A-pentacycle triterpene [24]. In our study, compounds **2**–**4** belong to A-ring contracted type triterpenes, which may derive from ursolic acid (**6**) and corresponding derivatives, and their intermediate products include compounds **7**–**12**. The plausible biosynthetic pathway of compounds **2**–**4** and **6**–**12** is shown in Figure 8, and the oleanane-type triterpenes (**1**, **5**, and **16**) also have a similar pathway. Meanwhile, aglycones are easily glycosylated at C-28 to form relevant triterpenoid saponins (**13**, **14**, **19**, and **20**) [25].

### 2.2. Biological Activities

The cytotoxic activities of all compounds (**1**–**23**) against RA-FLS cells and human cancer cell lines (Hep-G2, HCT-116, BGC-823, and MCF-7) were evaluated in vitro using the MTT method. As shown in Table 3, **6** and **7** showed good bioactivities against RA-FLS cells with IC_50_ values of 24.63 ± 1.96 and 25.12 ± 1.97 μM, respectively. Among the tested triterpenes, **4** and **19** exhibited a moderate cytotoxic effect on Hep-G2 cells (25.16 ± 2.55 and 17.66 ± 1.82 μM, respectively), and **16** and **21** were observed to inhibit HCT-116 cells (13.25 ± 1.65 and 21.62 ± 0.33 μM, respectively). Furthermore, **2** and **16** showed cytotoxic activities against BGC-823 with IC_50_ values of 24.76 ± 0.94 and 26.83 ± 2.52 μM, respectively. Except for the compounds mentioned above, other compounds with IC_50_ values higher than 30 μM were considered to be inactive against RA-FLS cells and human cancer cell lines.

## 3. Materials and Methods

### 3.1. General Information

Recording of 1D-NMR and 2D-NMR spectra was performed on a Bruker AV-600 spectrometer (Bruker, Billerica, MA, USA) with a single NMR probe at 600 MHz for ^1^H and 150 MHz for ^13^C in CD_3_OD. HR-ESI-MS experiments were performed using an Agilent 6200 series TOF/6500 series (Agilent, Santa Clara, CA, USA). A PerkinElmer Frontier MIR spectrometer (PerkinElmer, Waltham, MA, USA) was used to determine IR spectra. UV spectra were recorded on a PerkinElmer Lambda 650 (PerkinElmer, Waltham, MA, USA) in methanol. Optical rotations of compounds were determined by a Rudolph Research Analytical Autopol IV automatic polarimeter (Rudolph, Hackettstown, NJ, USA). An Applied Photophysics Chirascan plus CD spectrometer was used to determine ECD spectra. Semi-preparative RP-HPLC (Agilent, Palo Alto, CA, USA) was carried out on an Agilent 1100 system with an Agilent Eclipse XDB-C_18_ column (5 μm, 4.6 × 250 mm, Agilent, Palo Alto, CA, USA). Sephadex LH-20 for column chromatography was obtained from Pharmacia Fine Chemical Company, Ltd. (Uppsala, Sweden). Column chromatographic silica gel (80-100 and 200-300 mesh) and TLC plates (GF_254_) were purchased from Qingdao Marine Chemical Inc. (Qingdao, China). Acetonitrile and methanol (HPLC-grade) were obtained from Merck KGaA (Darmstadt, Germany). The other solvents were purchased from Shanghai Titan Scientific Co., Ltd. (Shanghai, China).

### 3.2. Plant Material

The dried roots of *P. freyniana* were collected at Huaihua of Hunan Province, China, in October 2019 and authenticated by Zai-Qi Zhang, a senior professor at Hunan University of Medicine. A voucher specimen (201910PF) was deposited at the TCM and Ethnomedicine Innovation and Development International Laboratory, School of Pharmacy, Hunan University of Chinese Medicine, Changsha, Hunan, People’s Republic of China.

### 3.3. Extraction and Isolation

The air-dried and pulverized roots of *P. freyniana* (10 kg) were extracted with 95% EtOH (10 L × 3, each 7 d). After concentration under reduced pressure, 600 g of crude EtOH extract was obtained. The EtOH extract was suspended in H_2_O and successively partitioned with PE (petroleum ether), CH_2_Cl_2_, EtOAc, and n-BuOH. Among them, the CH_2_Cl_2_-soluble fraction (41.5 g) was subjected to silica gel column chromatography (CC) eluted with PE/EtOAc (1:0–0:1) gradients to afford fourteen major fractions (C1–C14). Fraction C5 (587.4 mg) was fractioned on a Sephadex LH-20 column (CH_3_Cl/CH_3_OH, 1:1, *v*/*v*) to give three subfractions (C5.1–C5.3); then, subfraction C5.1 (387.4 mg) was isolated and purified by silica gel CC (gradient system, PE/EtOAc, 1:0–20:1, *v*/*v*) to obtain **18** (2.3 mg). Fraction C7 (1.2 g) was separated by a Sephadex LH-20 column with eluent (CH_3_Cl/CH_3_OH, 1:1, *v*/*v*) to give four subfractions (C7.1–C7.4). Subfraction C7.4 (38.8 mg) was chromatographed on a silica gel column (PE/EtOAc, 50:1–20:1, *v*/*v*) to give **23** (13.0 mg). Fraction C10 (1.2 g) was subjected to silica gel CC (PE/EtOAc, 20:1–8:1, *v*/*v*) after being separated on a Sephadex LH-20 column eluted with CH_3_Cl/CH_3_OH (1:1, *v*/*v*) to give nine subfractions (C10.1–C10.9). Subfraction C10.6 (99.6 mg) was purified by semi-preparative HPLC (CH_3_CN/CH_3_OH/H_2_O, 90:8:2) to obtain **1** (2.3 mg, t_R_ 9.8 min), **2** (8.3 mg, t_R_ 10.5 min), and **6** (36.0 mg, t_R_ 13.5 min). Subfraction C10.8 (140.5 mg) was purified by semi-preparative HPLC with the mobile phase (CH_3_CN/H_2_O, 87:13) after being separated with silica gel CC eluted with PE/EtOAc (20:1–8:1, *v*/*v*) gradients to yield **11** (1.0 mg, t_R_ 16.8 min). Fraction C11 (1.8 g) was chromatographed with a silica gel column (PE/EtOAc, 1:0–0:1, *v*/*v*) to give twelve subfractions (C11.1–C11.12), and subfraction C11.8.3 (233.6 mg) was purified by semi-preparative HPLC using CH_3_OH/CH_3_CN/H_2_O (40:40:20–45:45:10) as a gradient eluent to provide **5** (21.5 mg, t_R_ 14.7 min). Similarly, C11.10 (135.5 mg) was isolated by semi-preparative HPLC (CH_3_OH/H_2_O, 80:20–100:0) to give four further subfractions (C11.10.1–C11.10.4), and **7** (42.8 mg, t_R_ 11.6 min) was yielded by semi-preparative HPLC, eluting with the gradient mobile phase CH_3_OH/CH_3_CN/H_2_O (41:41:18–42:42:16) from subfraction C11.10.3 (38.1 mg). Then, the purification of **22** (2.9 mg, t_R_ 7.9 min) was conducted by semi-preparative HPLC (CH_3_CN/H_2_O, 90:10) from C11.10.4 (4.9 mg). Furthermore, subfraction C11.11 (485.7 mg) was isolated with silica gel CC (PE/EtOAc, 50:3, *v*/*v*) before being purified by semi-preparative HPLC using CH_3_CN/H_2_O (70:30–100:0) as the mobile phase to obtain **10** (0.9 mg, t_R_ 7.4 min). Fraction C12 (7.9 g) was subjected to silica gel CC using 2% dichloromethane in methanol as an isocratic eluent to afford five subfractions (C12.1–C12.5), and compound **12** (6.2 mg, t_R_ 6.4 min) was purified by semi-preparative HPLC (CH_3_OH/H_2_O, 82:18) from subfraction C12.4 (179.1 mg). C12.2 (3.0 g) was fractioned with silica gel CC (CH_2_Cl_2_/CH_3_OH, 49:1, *v*/*v*) to afford five subfractions (C12.2.1–C12.2.5). Among them, subfraction C12.2.3 (618.6 mg) was separated using silica gel CC (CH_2_Cl_2_/CH_3_OH, 99:1, *v*/*v*) and then by semi-preparative HPLC using 83% CH_3_OH in H_2_O as an isocratic eluent to yield **4** (34.1 mg, t_R_ 12.6 min). Subfraction C12.2.4 (408.3 mg) was further subjected to a Sephadex LH-20 column with eluent (CH_3_Cl/CH_3_OH,1:1, *v*/*v*) before being isolated by semi-preparative HPLC (CH_3_OH/H_2_O, 80:20–90:10) to give **8** (8.8 mg, t_R_ 8.6 min), **17** (3.0 mg, t_R_ 15.2 min), and two more subfractions (C12.2.4.1–C12.2.4.2). In addition, compounds **21** (5.0 mg, t_R_ 6.7 min) and **16** (4.5 mg, t_R_ 7.4 min) were obtained by semi-preparative HPLC (CH_3_CN/H_2_O, 98:2) from subfraction C12.2.4.2 (15.1 mg). Fraction C14 (11.2 g) was chromatographed with a silica gel column (CH_2_Cl_2_/CH_3_OH, 10:1, *v*/*v*) to afford three subfractions (C14.1–C14.3). Subfraction C14-1 (5.7 g) was further subjected to silica gel CC to obtain five subfractions (C11.14.1–C11.14.5), and C14.1.4 (871.5 mg) was purified by silica gel CC eluted with CH_2_Cl_2_/CH_3_OH (10:1, *v*/*v*) to yield **3** (261.4 mg). Then, C14.1.5 (207.3 mg) was isolated by semi-preparative HPLC (CH_3_CN/H_2_O 35:65–60:40) after being separated by silica gel CC (CH_2_Cl_2_/CH_3_OH, 47:3, *v*/*v*) to provide **20** (2.6 mg, t_R_ 7.3 min) and two further subfractions (C14.1.5.1-C14.1.5.2). C14.1.5.2 (5.0 mg) was isolated by semi-preparative HPLC using 28% CH_3_OH in H_2_O to yield **19** (2.5 mg, t_R_ 21.2 min) and **15** (1.8 mg, t_R_ 23.9 min). Similarly, compounds **9** (157.3 mg) and **14** (49.3 mg) were obtained by silica gel CC (CH_2_Cl_2_/CH_3_OH, 20:1, *v*/*v*) from subfraction C14.3 (1.5 g).

### 3.4. Characterization

2-hydroxymethyl-1-norolean-2,12-dien-29-oic acid **(1)**: White amorphous powder; ${\left[\alpha \right]}_{D}^{25}$ = +40.0 (*c* 0.1, MeOH); ECD (MeOH) Δε (nm): +26.2 (210) and -28.9 (220); IR (KBr) υ_max_: 3325, 2928, 2865, 1694, 1605, 1462, 1381, and 667 cm^−1^; for ^1^H- and ^13^C-NMR data, see Table 1; HR-ESI-MS (Pos.) *m*/*z* 477.3342 [M + Na]^+^ (calcd for C_30_H_46_NaO_3_, 477.3345).

2-hydroxymethyl-1-norursa-2,12-dien-29-oic acid **(2)**: White amorphous powder; ${\left[\alpha \right]}_{D}^{25}$ = +50.0 (*c* 0.1, MeOH); ECD (MeOH) Δε (nm): +66.3 (203) and −11.7 (223); IR (KBr) υ_max_: 3362, 2964, 2860, 1687, 1585, 1459, 1380, and 1023 cm^−1^; for ^1^H- and ^13^C-NMR data, see Table 1; HR-ESI-MS (Pos.) *m*/*z* 477.3361 [M + Na]^+^ (calcd for C_30_H_46_NaO_3_, 477.3345).

17-(4′-hydroxy-3′-methoxybenzoate)-kaur-16-en-19-oic acid **(22)**: White amorphous powder; ${\left[\alpha \right]}_{D}^{25}$ = −80.0 (*c* 0.1, MeOH); UV (MeOH) λ_max_(log ε) = 199 (4.5) nm; ECD (MeOH) Δε (nm): +19.6 (209) and −27.2 (223); IR (KBr) υ_max_: 3406, 2921, 1698, 1280, and 1030 cm^−1^; for ^1^H- and ^13^C-NMR data, see Table 2; HR-ESI-MS (Pos.) *m*/*z* 493.2556 [M + Na]^+^ (calcd for C_28_H_38_NaO_6_493.2566).

### 3.5. Cell Culture

The human RA-FLS cell line and human cancer cell lines (Hep-G2, HCT-116, BGC-823, and MCF-7) were purchased from Shanghai Fu-Heng Biological Technology Co., Ltd. (Shanghai, China). RA-FLS cells were cultured in DMEM/F-12 with 10% fetal bovine serum (FBS); the Hep-G2, BGC-823, and MCF-7 cell lines in DMEM with 10% FBS; and the HCT-116 cell line in McCoy’s 5A with 10% FBS. Cell cultures were maintained at 37 °C in a humidified, 5% CO_2_ atmosphere [26].

### 3.6. Cytotoxicity Assessment

The cytotoxicity activities were evaluated by the MTT assay [26]. Firstly, the tested compounds (**1**–**23**) were diluted in corresponding media from freshly made solutions in DMSO (50 mM) to working concentrations (0–40 μM). The cells were seeded in 96-well plates. After 24 h incubation, the cells were exposed to the test compounds at the indicated concentrations for 24 h under 5% CO_2_ at 37 °C. Then, 100 μL of MTT (0.5 mg/mL) was added to each well and incubated for 4 h. Subsequently, the supernatant was removed from the formazan crystals, and 100 μL DMSO was added to each well. Finally, the absorbance was measured by a microplate reader using a wavelength of 492 nm to determine cell viability rate.

## 4. Conclusions

As a common Dong ethnomedicine in China, the roots of *P. freyniana* have been widely used for heat clearing and detoxifying. Moreover, ursane-type triterpenes are some of the most common pentacyclic triterpenes in ethnic medicine, with various pharmacological activities and low toxicity [27]. In this work, twenty-three compounds, comprising two unusual A-ring contracted triterpenoids named madengaisu A and B (**1** and **2**) and one new *ent*-kaurane diterpenoid named madengaisu C (**22**) along with 20 known compounds, were isolated and identified, thirteen of which were classified as ursane-type triterpenoids. In addition, this article is the first to report the diterpenoid constituents from the genus *Potentilla* L. Compounds **5**, **11**, **15**, **16**, **19**, **22**, and **23** were isolated from the genus *Potentilla* L. for the first time, and compounds **4**, **6**, **7**, **10**, and **20** were isolated from this plant for the first time, which enriched the chemical metabolite diversity of diterpenoids and triterpenoids in this plant. Additionally, compounds **2**, **4**, **6**, **7**, **16**, **19**, and **21** showed good anti-RA and/or anti-tumor activities, which demonstrated that triterpenes from *P. freyniana* may serve as therapeutic agents for RA and cancer treatment.

## Figures and Tables

**Table 1:** The ^1^H and ^13^C-NMR data of **1** and **2** (600/150 MHz, CD_3_OD, *δ* in ppm, *J* in Hz).

Position	1		2	
	*δ*_C_	*δ*_H_	*δ*_C_	*δ*_H_
1	61.4	4.16, dd (14.5, 1.8) 4.09, dd (14.5, 1.7)	61.4	4.16, dd (14.5, 1.8) 4.08, dd (14.5, 1.7)
2	156.0	-	156.1	-
3	135.4	5.40, s	135.5	5.39, s
4	43.3	-	43.3	-
5	64.5	1.44, m	64.4	1.41, m
6	18.2	1.52, m	18.2	1.49, m
7	34.9	a 1.56, m b 1.38, m	35.4	a 1.58, m b 1.39, m
8	42.1	-	42.3	-
9	44.9	2.15, m	44.8	2.09, m
10	51.8	-	51.7	-
11	27.5	b 2.19, m a 2.03, m	27.4	b 2.18, m a 2.07, m
12	123.6	5.25, t (3.5)	126.8	5.22, t (3.5)
13	145.7	-	140.1	-
14	43.2	-	43.5	-
15	29.1	b 1.83, dt (13.8, 4.3) a 1.09, dt (13.7, 3.4)	29.5	b 1.95, td (13.6, 4.5) a 1.08, ddd (13.7, 4.3, 2.4)
16	24.0	a 2.05, m b 1.61, ddt (13.6, 4.2, 2.2)	25.3	a 2.05, m b b 1.65, m a
17	47.7	-	(48.6) *	-
18	43.0	2.85, dd (13.9, 4.6)	54.5	2.20, m
19	47.0	a 1.72, m	40.2	1.38.m
20	31.6	-	40.4	0.97, overlapped
21	35.1	a 1.42, m b 1.23, m	31.7	1.51, m 1.36, m
22	33.8	b 1.77, m a 1.55, m	38.1	1.68, m 1.63, m
23	30.3	1.04, s	30.3	1.03, s
24	21.8	0.96, s	21.8	0.95, s
25	19.3	1.16, s	19.3	1.16, s
26	19.2	0.87, s	19.3	0.90, s
27	26.8	1.20, s	24.3	1.15, s
28	181.9	-	181.7	-
29	33.6	0.91, s	17.7	0.90, d (6.6)
30	24.0	0.95, s	21.6	0.96, d (4.8)

a: *α*-oriented proton. b: *β*-oriented proton. * Overlapped with the solvent peak.

**Table 2:** The ^1^H and ^13^C-NMR data of compound **22** (600/150 MHz, CD_3_OD, *δ* in ppm, *J* in Hz).

Position	22		Position	22	
	*δ*_C_	*δ*_H_		*δ*_C_	*δ*_H_
1	42.2	1.91, m 0.86, m	15	46.2	1.67, overlapped 1.08, m
2	20.4	1.92, m 1.30, m	16	41.2	2.26, m
3	39.3	2.13, overlapped 1.01, m	17	69.9	4.09, d (1.7) 4.08, d (1.7)
4	44.8	-	18	29.6	1.19, s
5	58.3	1.07, overlapped	19	182.4	-
6	23.7	1.87, m	20	16.3	0.99, s
7	42.9	1.50, m	1′	122.8	-
8	46.2	-	2′	113.5	7.54, d (2.0)
9	56.8	1.07, overlapped	3′	148.8	-
10	40.8	-	4′	152.9	-
11	19.8	1.69, overlapped	5′	116.0	6.85, d (8.8)
12	32.5	1.60, m 1.48, m	6′	125.0	7.55, dd (2.0 8.7)
13	40.2	2.16, m	7′	168.4	-
14	38.3	1.95, m 1.16, m	-OCH_3_	56.4	3.90, s

**Table 3:** Cytotoxic effects of tested compounds on RA-FLS and human cancer cell lines.

Compound	IC_50_ (μM) ^a^				
	Anti-RA-FLS Activity	Anti-Tumor Activity			
	RA-FLS	Hep-G2	HCT-116	BGC-823	MCF-7
**2**	>30 ^b^	>30	>30	24.76 ± 0.94	>30
**4**	>30	25.16 ± 2.55	>30	>30	>30
**6**	24.63 ± 1.96	>30	>30	>30	>30
**7**	25.12 ± 1.97	>30	>30	>30	>30
**16**	>30	>30	13.25 ± 1.65	26.83 ± 2.52	>30
**19**	>30	17.66 ± 1.82	>30	>30	>30
**21**	>30	>30	21.62 ± 0.33	>30	>30
Methotrexate ^c^	5.09 ± 0.60	-	-	-	-
Taxol (nM) ^c^	-	13.82 ± 0.78	15.79 ± 0.91	9.12 ± 1.23	-

^a^ IC_50_, the half maximal inhibitory concentration. Values represent the mean ± SD of three independent experiments. ^b^ Compounds exhibiting IC_50_ values > 30 μM were considered to be inactive. ^c^ Methotrexate as positive control for anti-RA-FLS activity test and taxol for anti-tumor activity test.

## Data Availability

The raw data that support the findings of this study are available from the corresponding authors (J.W., L.B., and W.W.) upon reasonable request.

## Supplementary Materials

The following supporting information can be downloaded at: https://www.mdpi.com/article/10.3390/molecules27123665/s1, Figures S1–S26: UV, IR, HR ESIMS and NMR spectra of compound **1**–**2** and **22**.; Tables S1–S6: ECD calculations results of compound **1**–**2** and **22**. Refs. [28,29,30] are cited in Supplementary Materials.

## Sample Availability

Samples of the compounds **1**–**23** are available from the authors.
