# Supplementary material for: New Terpenoids from Potentilla freyniana Bornm. and Their Cytotoxic Activities
Source: Molecules. 2022 Jun 7;27(12):3665. doi: 10.3390/molecules27123665 (PMC9227482; doi:10.3390/molecules27123665)
Supplement: Supplementary file 1 [file molecules-27-03665-s001.zip › molecules-1753005-supplementary.pdf]

SUPPLEMENTARY MATERIAL

New Terpenoids from *Potentilla freyniana* Bornm. and Their Cytotoxic Activities

Jia Wu [1, †], Zai-Qi Zhang [2, †], Xu-Dong Zhou[1], Qing-Ying Yao[1], Zhu-Liang Chen [1], Ling-Ling Chu [1], Huang-He Yu [1], Yu-Pei Yang [1], Bin Li [1, *] and Wei Wang [1, *]

1 TCM and Ethnomedicine Innovation and Development International Laboratory, School of Pharmacy, Hunan University of Chinese Medicine, Changsha 410208, China; a17607310485@163.com (J.W.); xudongzhou999@163.com (X.-D.Z.); yaoqyyqy@163.com (Q.-Y.Y.); a844135048@163.com (Z.-L.C.); 13101112670@163.com (L.-L.C); yhh@hnucm.edu.cn (H.-H.Y.); yang-yupei24@163.com (Y.-P.Y.)

2 Hunan Provincial Key Laboratory of Dong Medicine, Hunan University of Medicine, Huaihua 418000, China; qizaizhang@126.com

* Correspondence: wangwei402@hotmail.com(W.W.); libin@hnucm.edu.cn (B.L.); Tel.: (0731)88458227(W.W.)

† These authors contributed equally to this work

1. Experimental Section

1.1 ECD calculations of compounds 1-2, 22

In general, conformational analyses were carried out via random searching in the Sybyl-X 2.0 using the MMFF94S force field with an energy cutoff of 5 kcal/mol. [1] The results showed one lowest energy conformers. Subsequently, geometry optimizations and frequency analyses were implemented at the B3LYP-D3(BJ)/6-31G* level in PCM methanol using ORCA5.0.1[2] All conformers used for property calculations in this work were characterized to be stable point on potential energy surface (PES) with no imaginary frequencies. The excitation energies, oscillator strengths, and rotational strengths (velocity) of the first 60 excited states were calculated using the TD-DFT methodology at the PBE0/def2-TZVP level in PCM methanol using ORCA5.0.1.[2] The ECD spectra were simulated by the overlapping Gaussian function (half the bandwidth at 1/e peak height, sigma = 0.30 for all).[3] Gibbs free energies for conformers were determined by using thermal correction at B3LYP-D3(BJ)/6-31G* level and electronic energies evaluated at the wB97M-V/def2-TZVP level in PCM methanol using ORCA5.0.1[2] To get the final spectra, the simulated spectra of the conformers were averaged according to the Boltzmann distribution theory and their relative Gibbs free energy (ΔG). By comparing the experiment spectra with the calculated model molecules, the absolute configuration of the only chiral center was determined to be.

[1]. Sybyl Software, version X 2.0; Tripos Associates Inc.: St. Louis, MO, 2013.

[2]. Neese, F. (2012) The ORCA program system, Wiley Interdiscip. Rev.: Comput. Mol. Sci., 2, 73-78.

[3]. Stephens, P. J.; Harada, N. ECD cotton effect approximated by the Gaussian curve and other methods. Chirality2010, 22, 229–233.

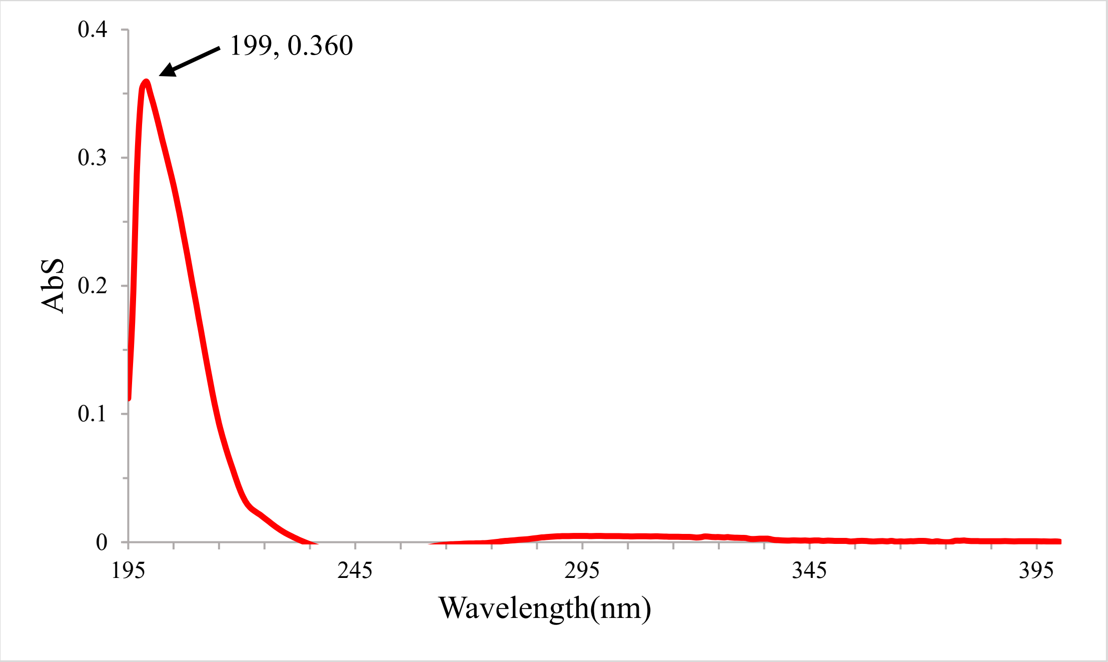

Figure S1. UV Spectrum of compound **1** in MeOH.

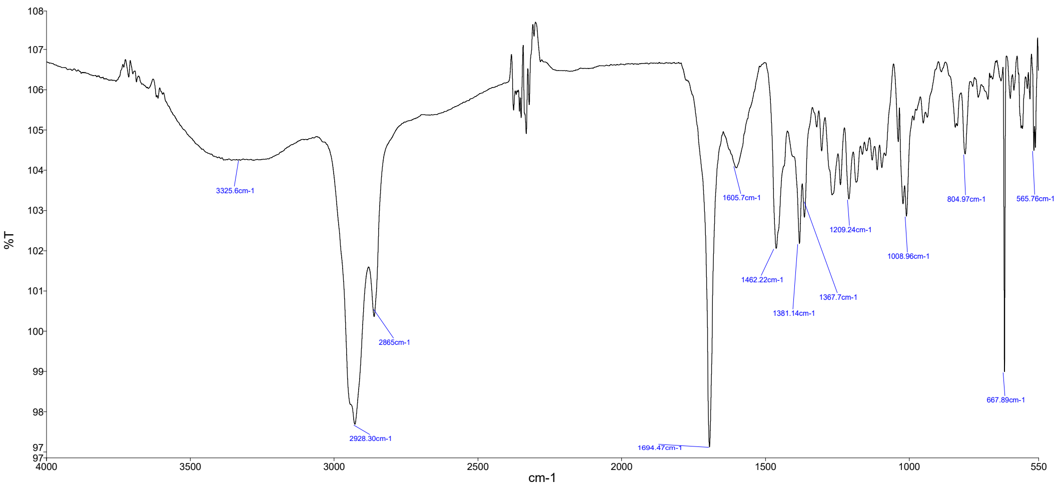

Figure S2. IR Spectrum (KBr) of compound **1**.

Qualitative Analysis Report

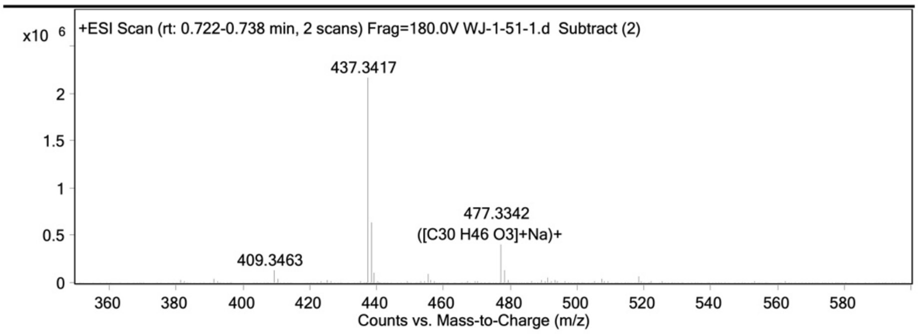

Figure S3. HR ESIMS spectrum of compound **1**.

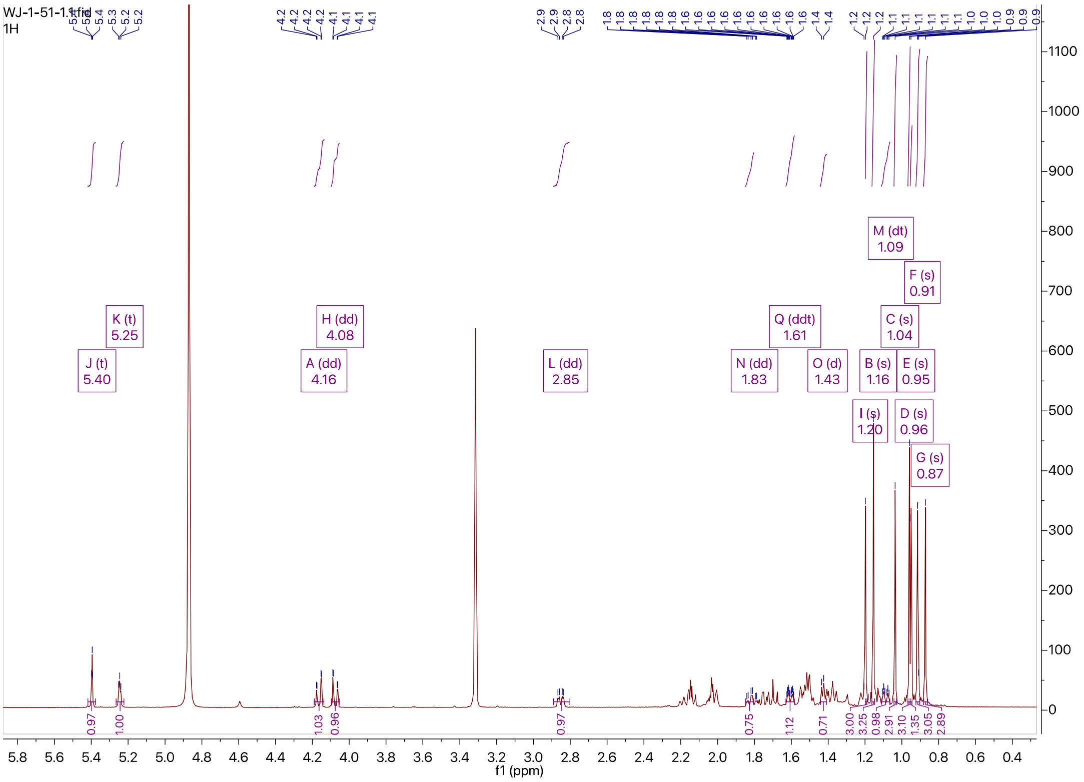

Figure S4. ^{1}H NMR spectrum (600MHz, CD_3OD) of compound **1**.

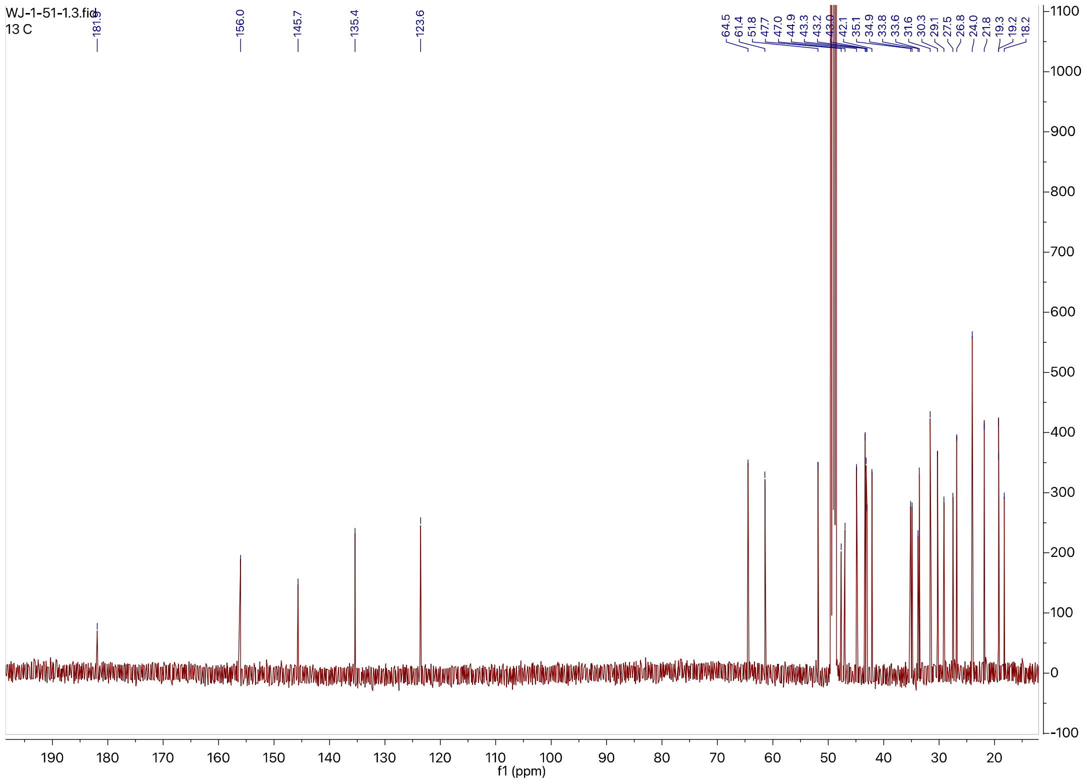

Figure S5. ^{13}C NMR spectrum (150MHz, CD_3OD) of compound **1**.

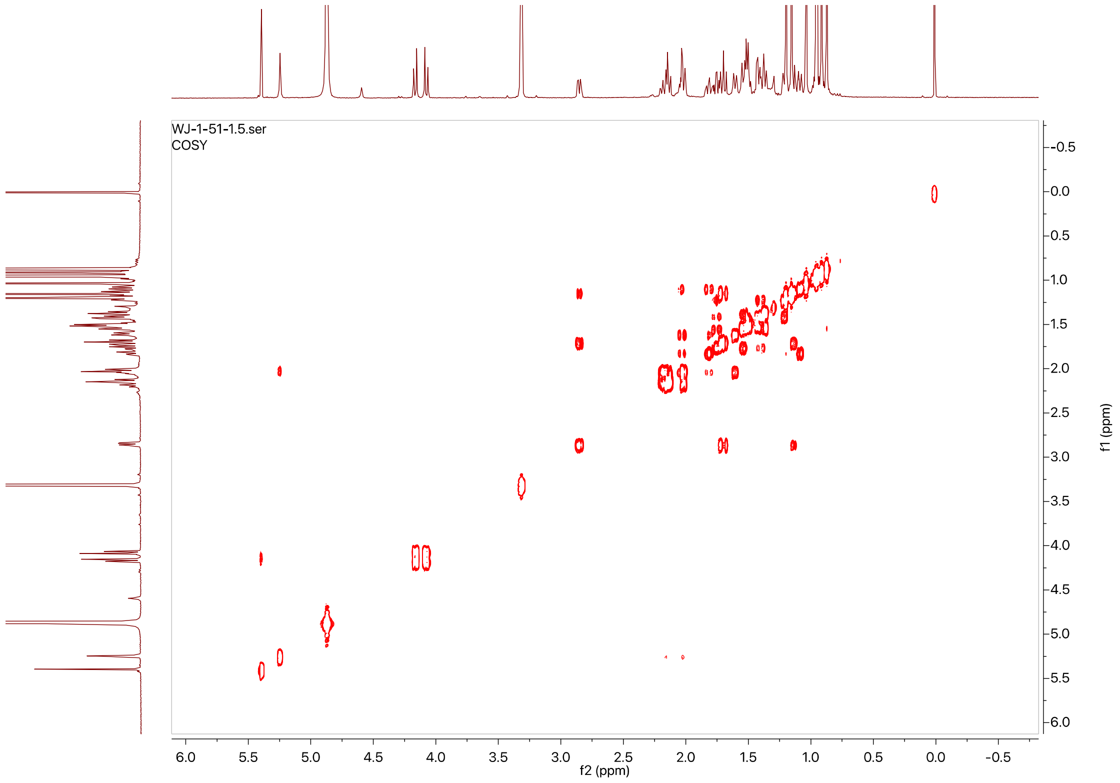

Figure S6. ^{1}H-^{1}H COSY spectrum (CD_3OD) of compound **1**.

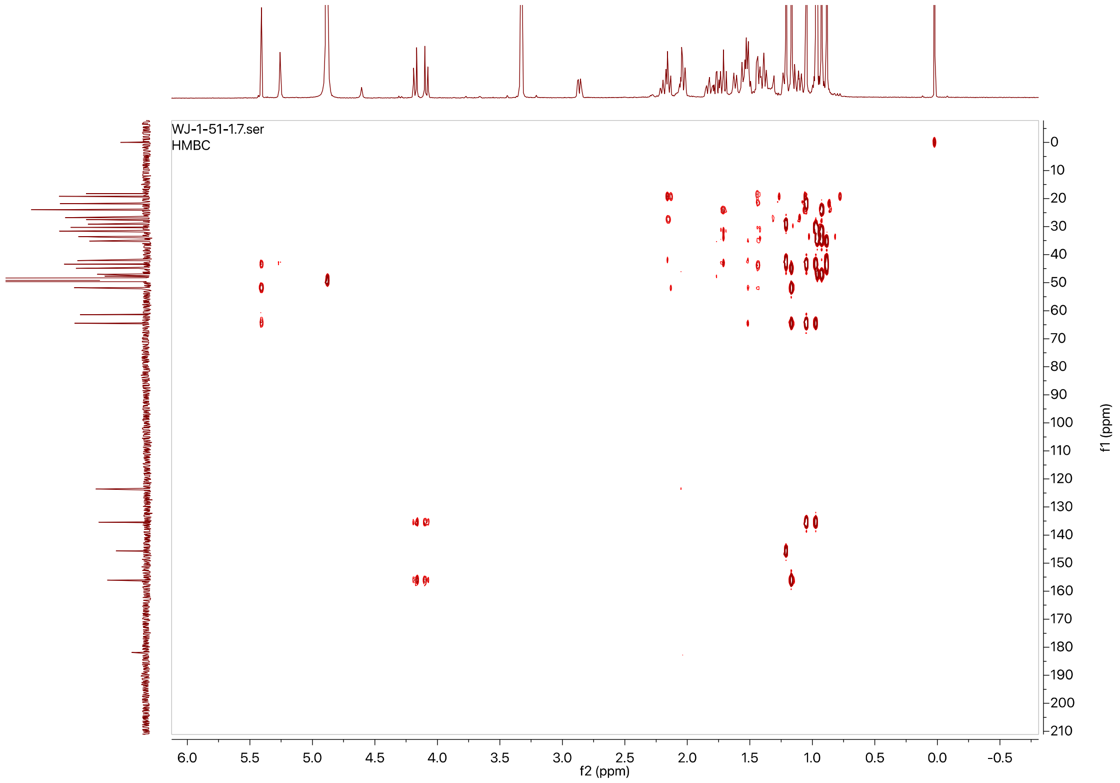

Figure S7. HMBC spectrum (CD_3OD) of compound **1**.

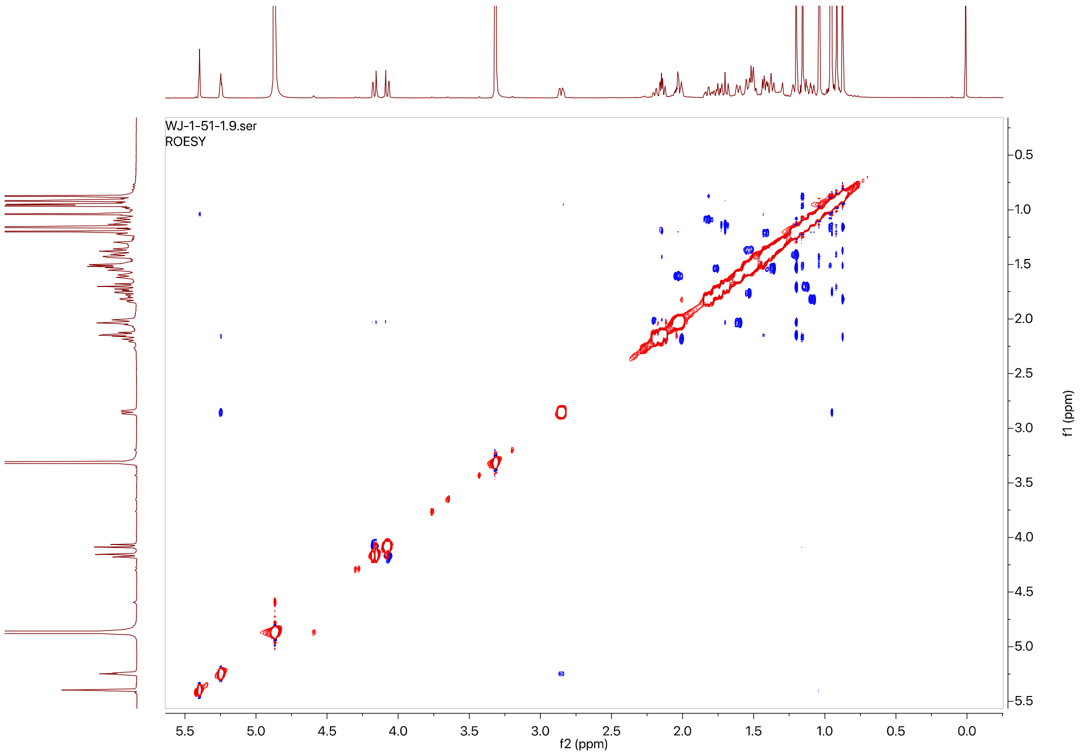

Figure S8. ROESY spectrum (CD_3OD) of compound **1**.

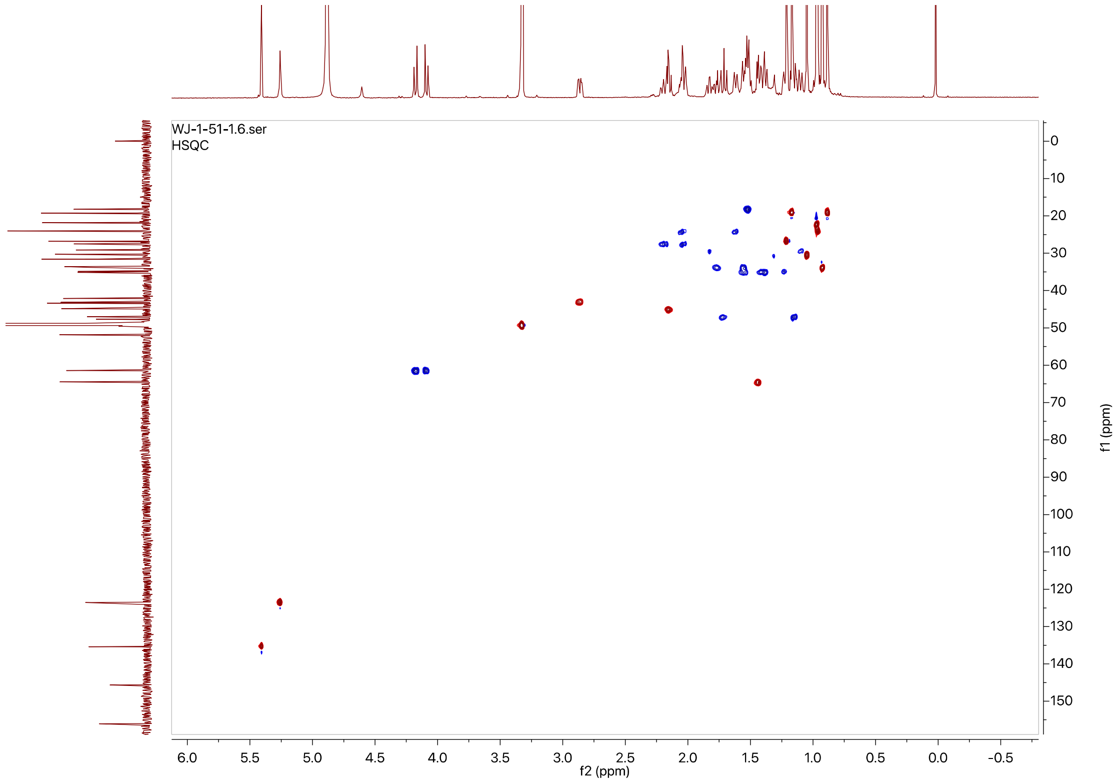

Figure S9. HSQC spectrum (CD_3OD) of compound **1**.

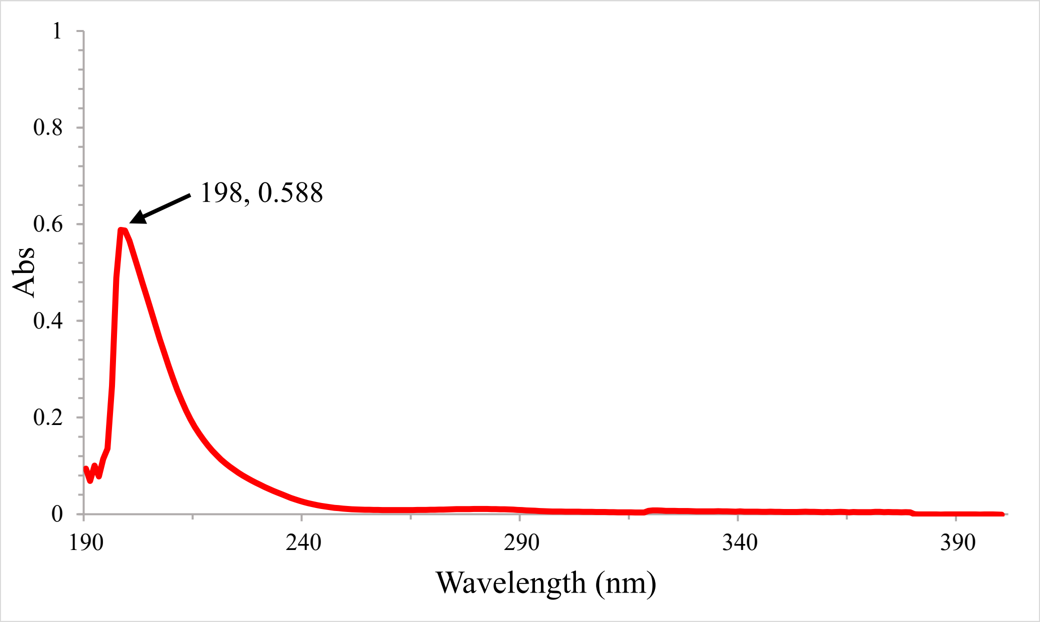

Figure S10. UV Spectrum of compound **2** in MeOH.

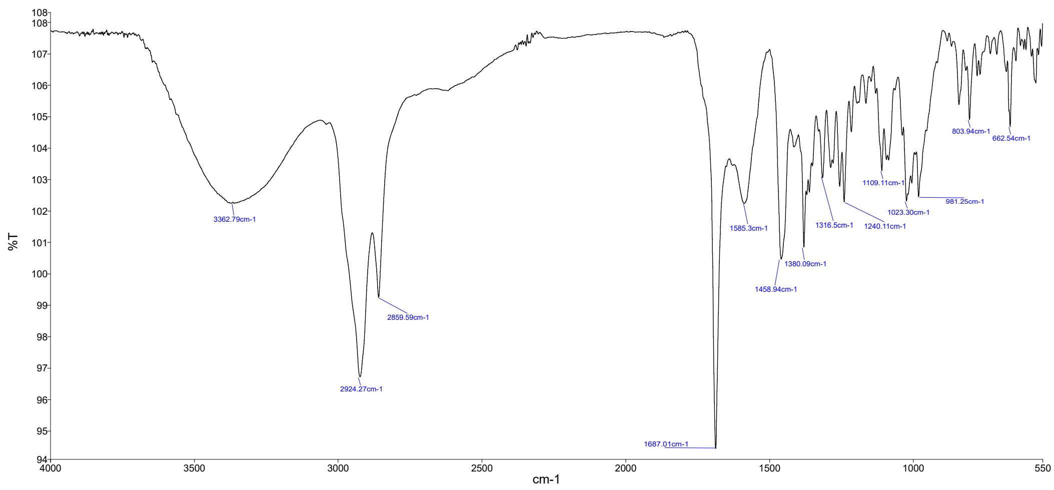

Figure S11. IR Spectrum (KBr) of compound **2**.

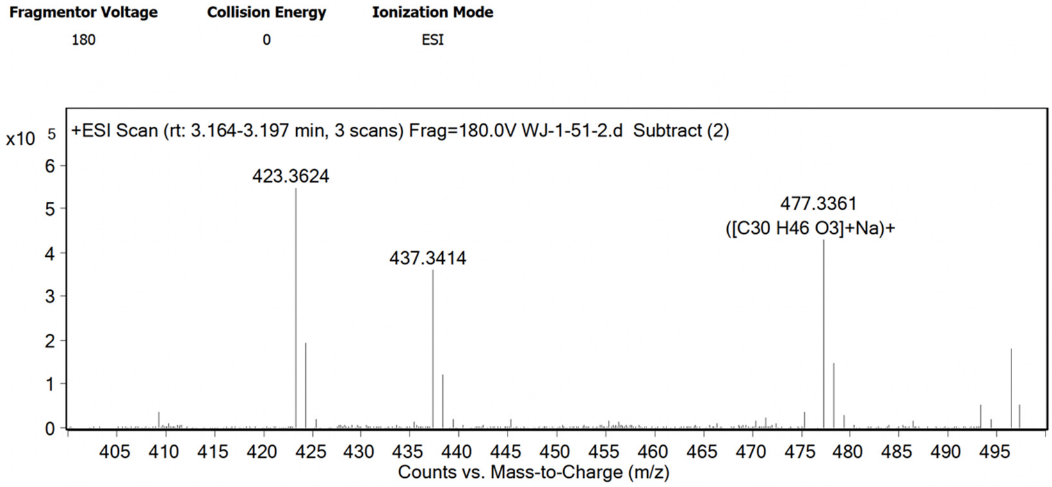

Figure S12. HR ESIMS spectrum of compound **2**.

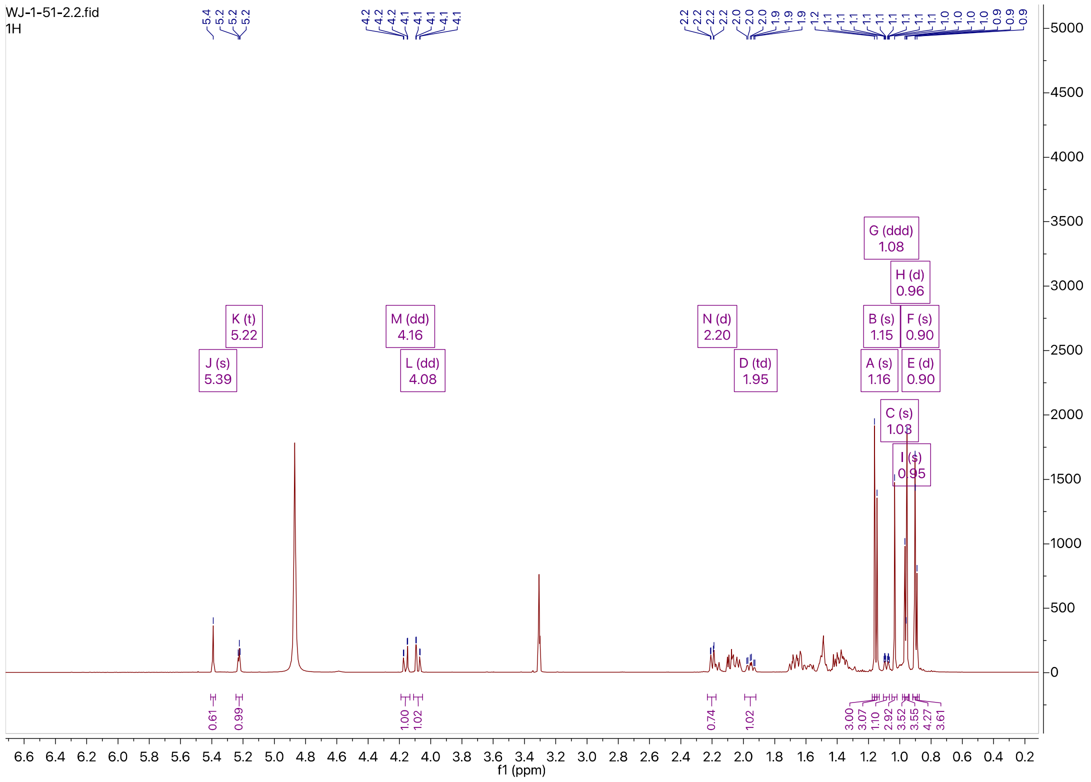

Figure S13. ^{1}H NMR spectrum (600MHz, CD_3OD) of compound **2**.

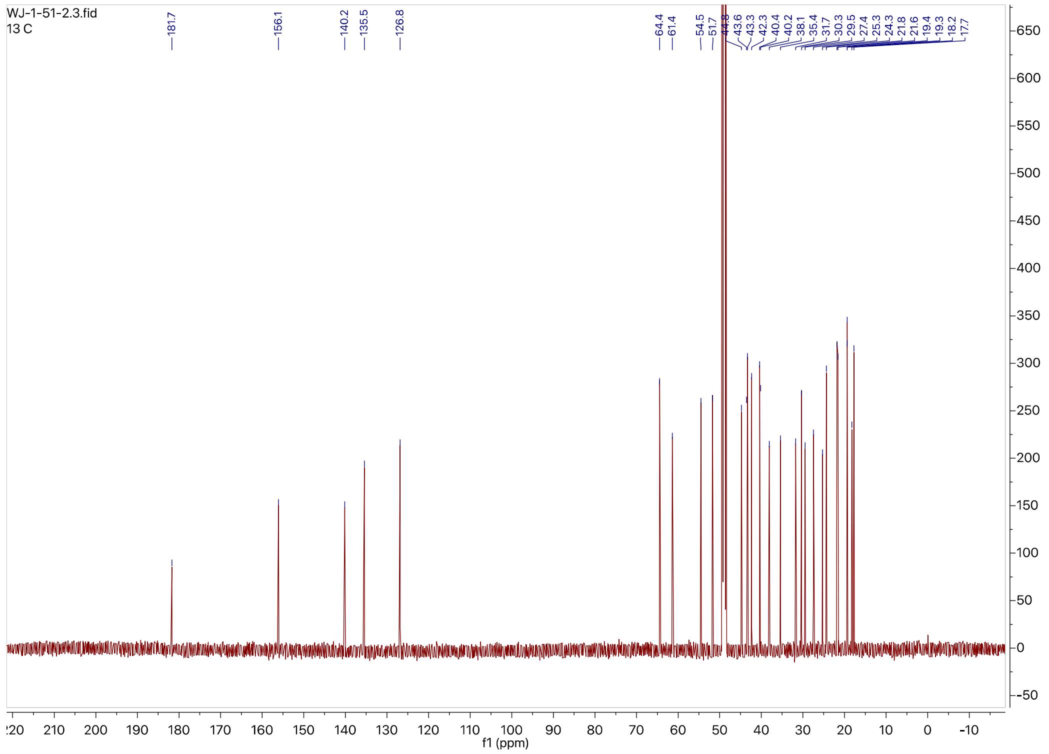

Figure S14. ^{13}C NMR spectrum (150MHz, CD_3OD) of compound **2**.

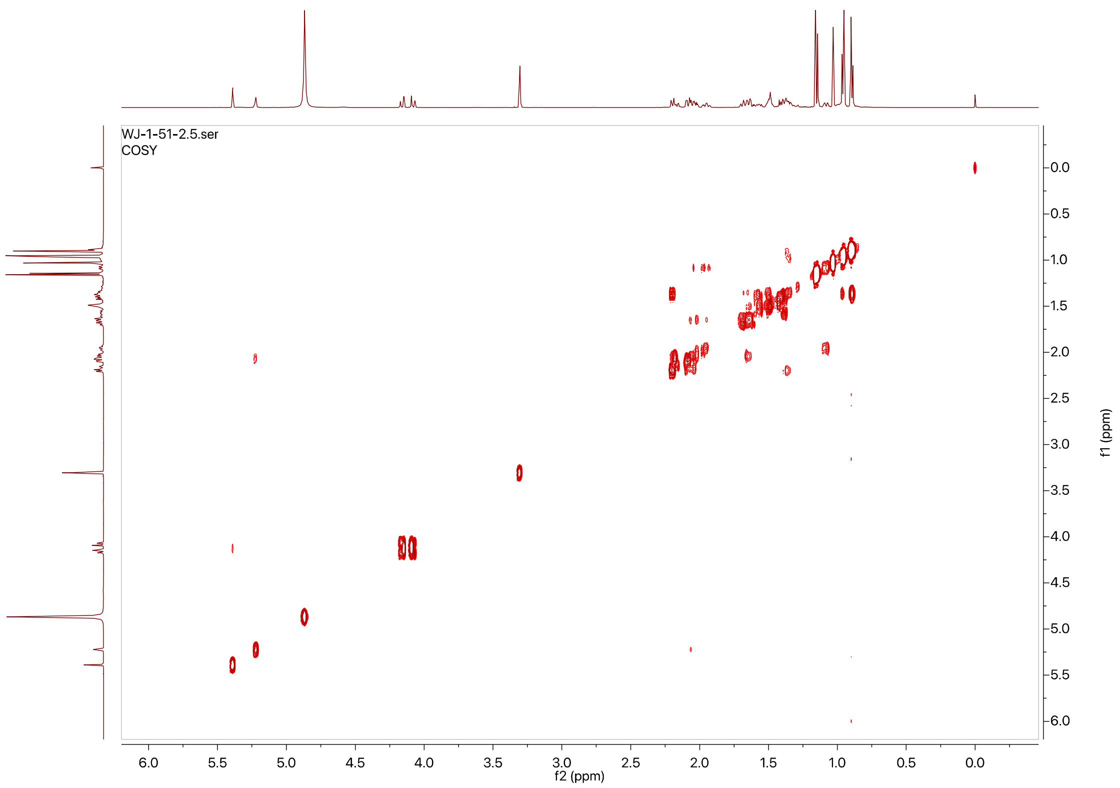

Figure S15. ^{1}H-^{1}H COSY spectrum (CD_3OD) of compound **2**.

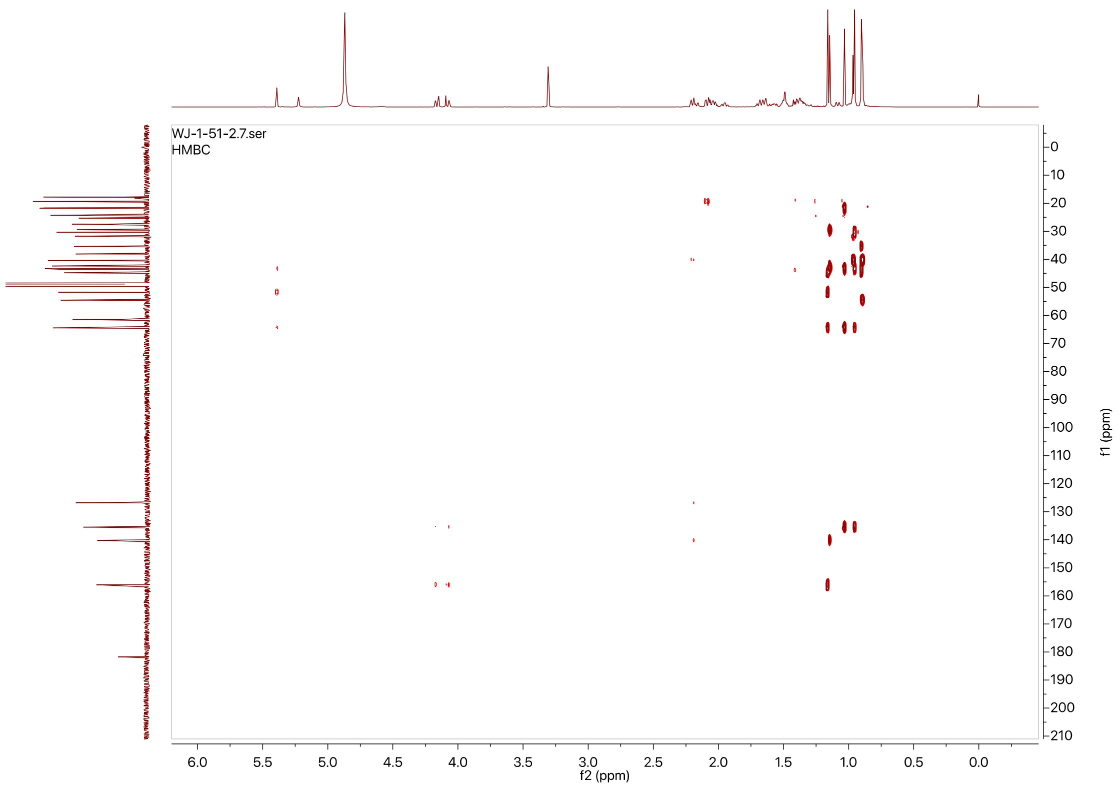

Figure S16. HMBC spectrum (CD_3OD) of compound **2**.

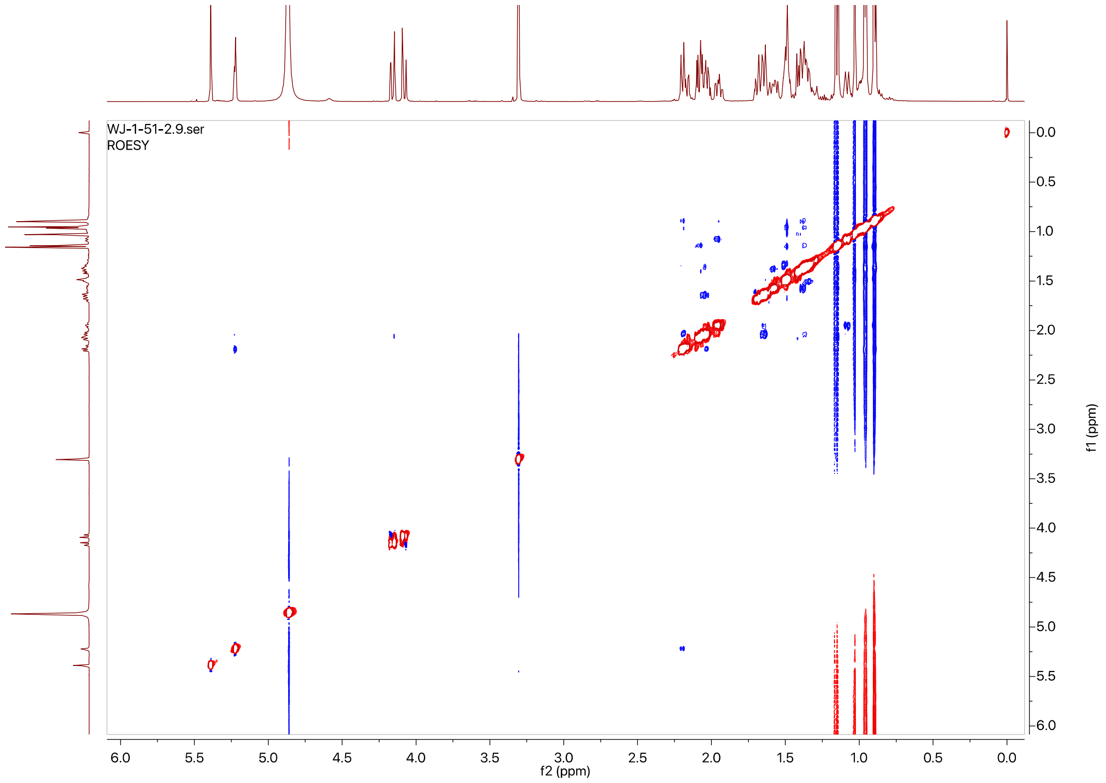

Figure S17. ROESY spectrum (CD_3OD) of compound **2**.

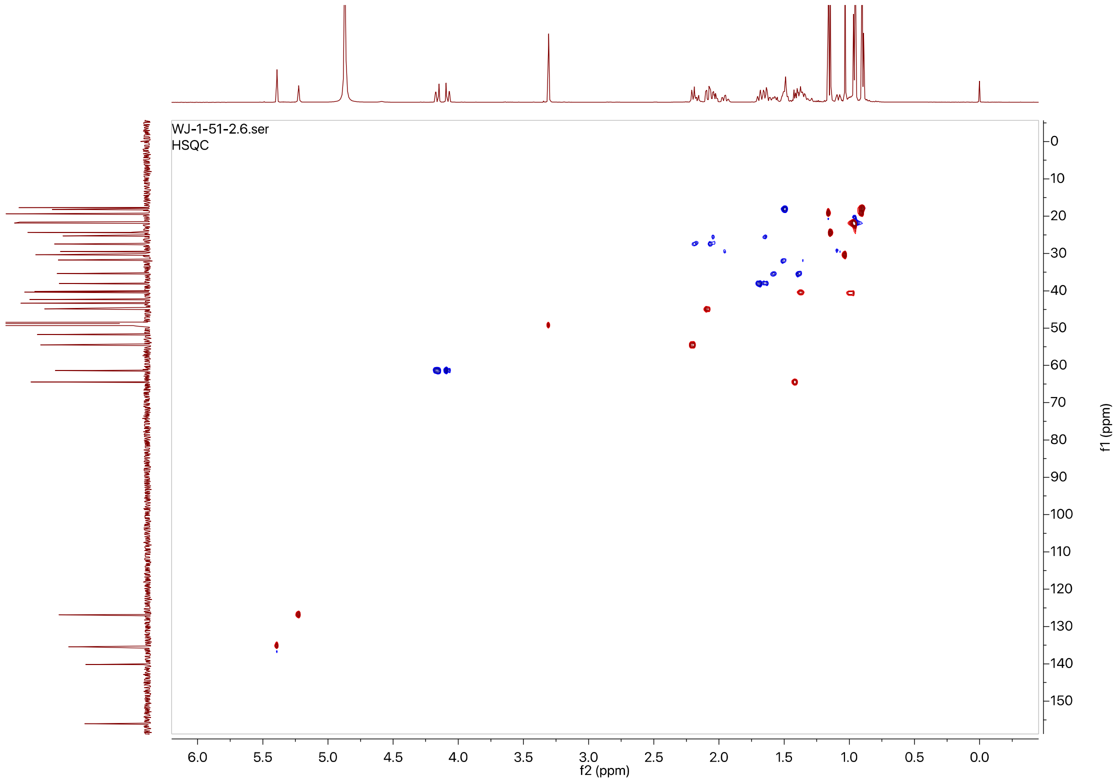

Figure S18. HSQC spectrum (CD_3OD) of compound **2**.

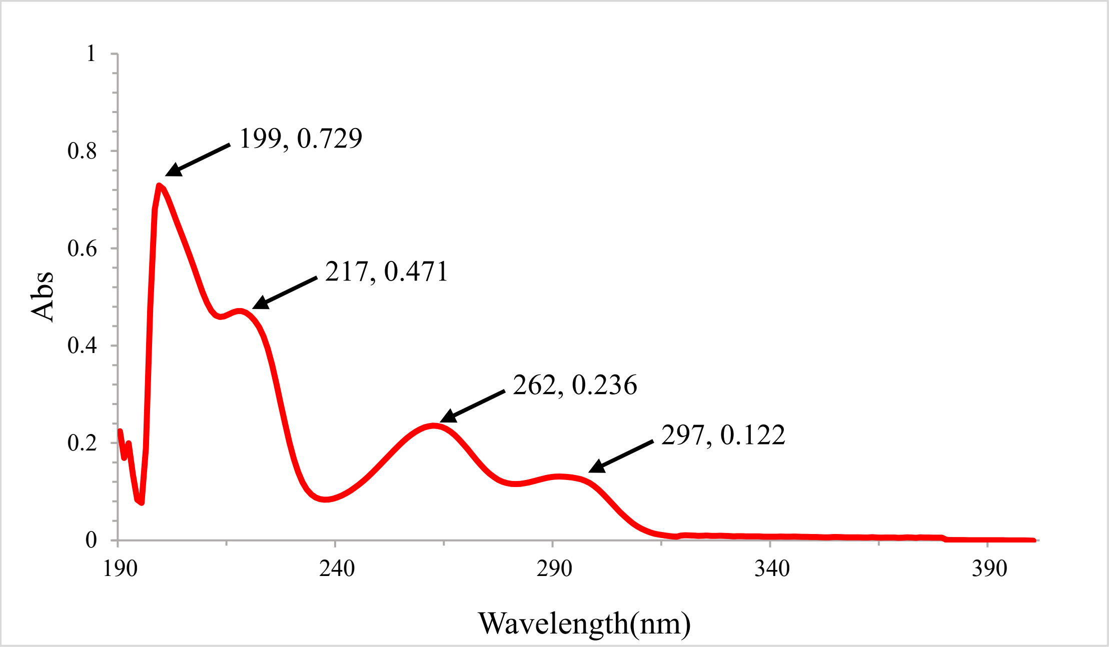

Figure S19. UV Spectrum of compound **22** in MeOH.

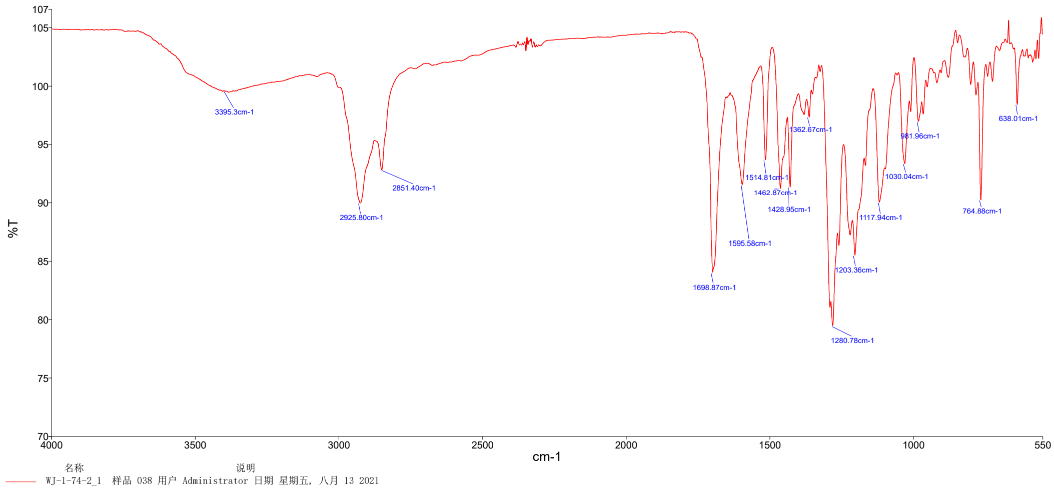

Figure S20. IR Spectrum (KBr) of compound **22**.

Fragmentor Voltage	Collision Energy	Ionization Mode
180	0	ESI

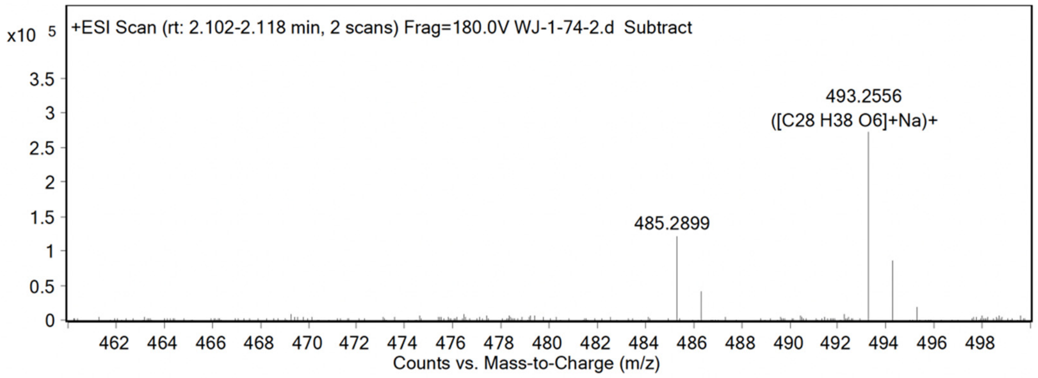

Figure S21. HR ESIMS spectrum of compound **22**.

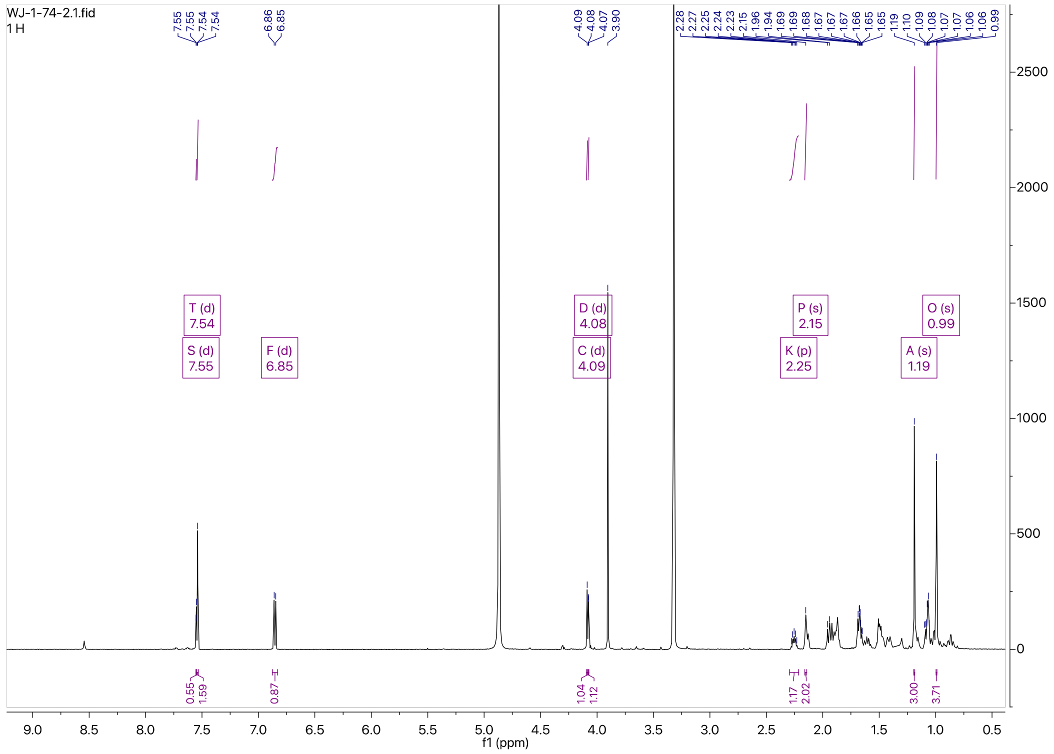

Figure S22. ^{1}H NMR spectrum (600MHz, CD_3OD) of compound **22**.

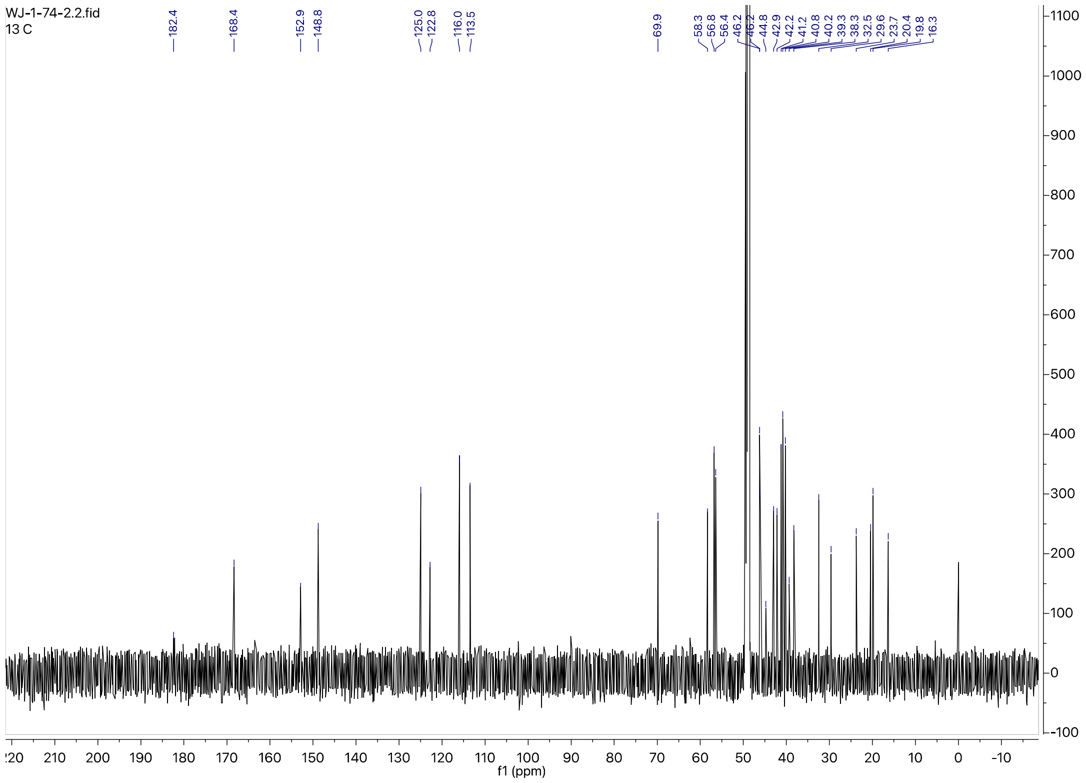

Figure S23. ^{13}C NMR spectrum (150MHz, CD_3OD) of compound **22**.

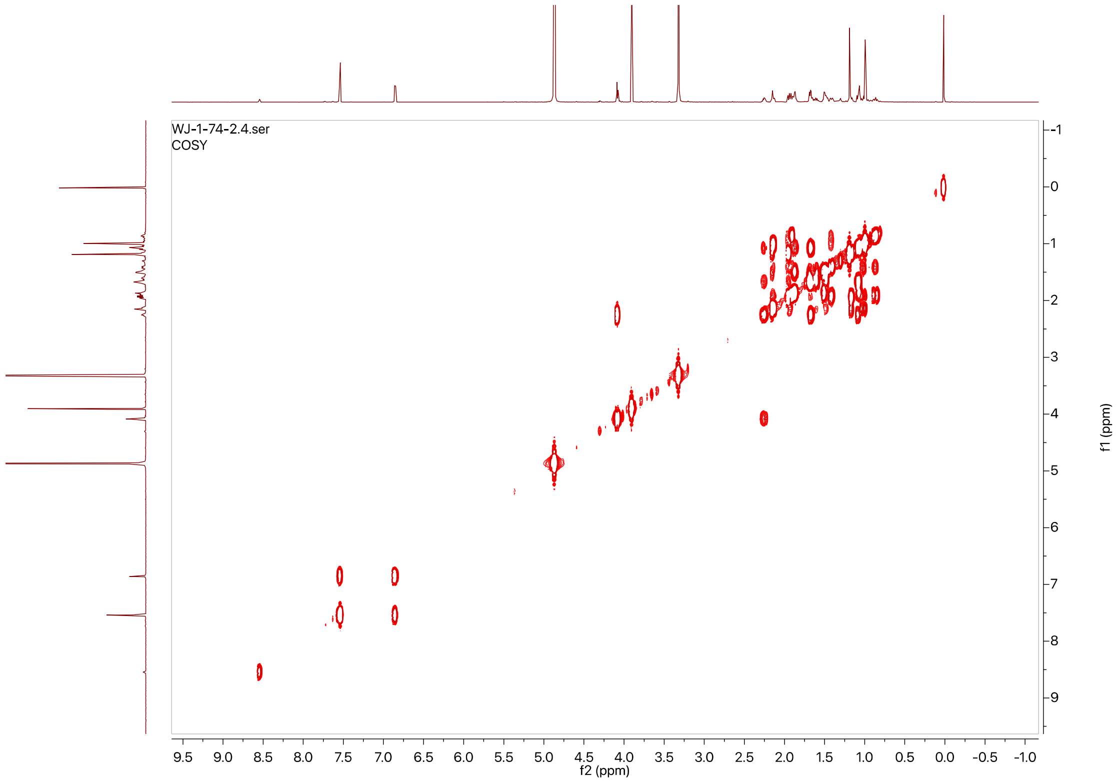

Figure S24. ^{1}H-^{1}H COSY spectrum (CD_3OD) of compound **22**.

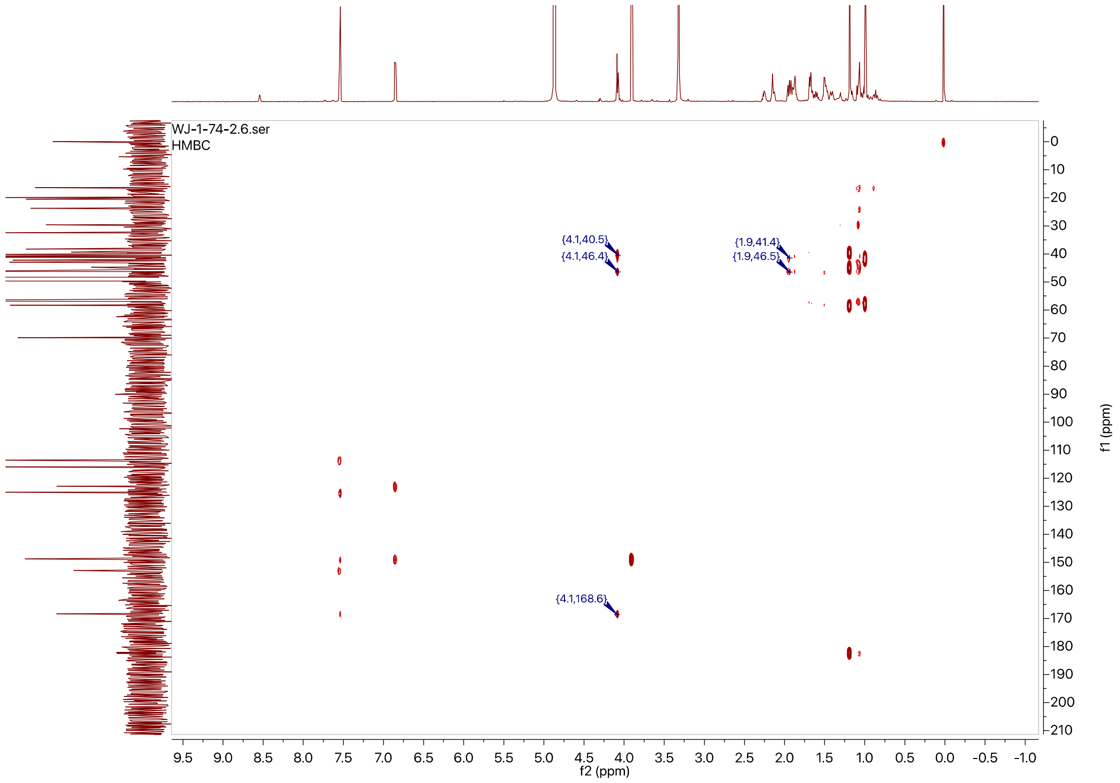

Figure S25. HMBC spectrum (CD_3OD) of compound **22**.

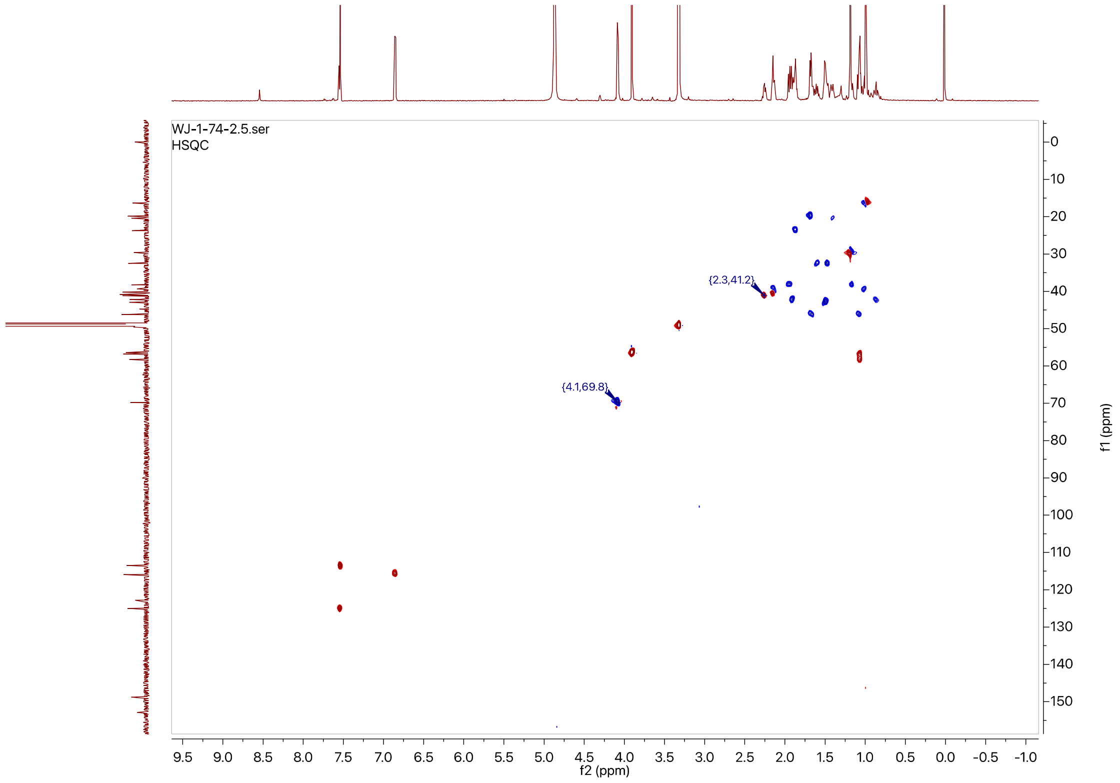

Figure S26. HSQC spectrum (CD_3OD) of compound **22**.

Table S1 Gibbs free energies[a] and equilibrium populations[b] of low-energy conformers of compound **1**.

Conformers	ΔG(a.u.)	P(%)/100	G(a.u.)
C1000001_tddft_	0.0000	100.0	-1395.870956

[a]wB97M-V/def2-TZVP, in a.u.

[b]From ΔG values at 298.15K.

Table S2 Cartesian coordinates for the low-energy reoptimized random reseach conformers of compound **1** at B3LYP-D3(BJ)/6-31G* level of theory in methanol.

C000001_en_		Standard Orientation (A.U.)			
Center number	Atomic number	Atomic Type	X	Y	Z
0	6	0	1.7649	1.744613	0.582557
1	6	0	-5.379746	-2.174271	-1.34664
2	6	0	1.830083	-1.151696	0.438593
3	6	0	4.308609	3.075851	0.800512
4	6	0	-0.914681	-2.300112	0.876051
5	6	0	6.057457	1.956282	2.865449
6	6	0	-2.792991	-0.833672	-0.905209
7	6	0	-4.736444	-4.916997	-2.149599
8	6	0	5.168366	2.302092	5.59234
9	6	0	4.206238	5.970144	0.900743
10	8	0	2.803953	3.257005	5.855116
11	6	0	-0.385682	3.047937	0.396912
12	6	0	3.672201	-2.164511	2.484723
13	6	0	-0.870077	-5.187488	0.345376
14	6	0	-2.938918	1.914729	-0.036306
15	6	0	6.266318	-0.914164	2.430892
16	6	0	-3.470675	-6.413013	-0.066376
17	6	0	8.689591	3.183842	2.660732
18	8	0	6.434724	1.733028	7.410716
19	6	0	6.825671	7.250895	0.830383
20	6	0	8.545132	6.058379	2.850244
21	6	0	-7.232843	-1.991748	0.926586
22	6	0	2.918881	-1.920219	-2.174182
23	6	0	-1.697863	-1.921704	3.664905
24	6	0	-6.898887	-1.385622	-3.704034
25	6	0	-7.868842	-3.42885	-4.836955
26	6	0	-7.001149	-5.910779	-3.701049
27	6	0	-6.125501	-7.759644	-5.757714
28	6	0	-9.072145	-7.203183	-2.124702
29	1	0	-3.265165	-4.655107	-3.605146

30	1	0	-1.925294	-0.785734	-2.791369
31	1	0	5.320196	2.606561	-0.959222
32	6	0	8.023239	7.016072	-1.803963
33	6	0	6.491117	10.068564	1.417698
34	6	0	-7.450888	1.270455	-4.501205
35	8	0	-8.887488	2.687662	-2.686676
36	1	0	3.102471	6.671247	-0.709786
37	1	0	3.215054	6.571744	2.618705
38	1	0	2.494113	3.328098	7.663899
39	1	0	-0.351495	5.093936	0.572199
40	1	0	2.872132	-1.864831	4.368267
41	1	0	3.88457	-4.21178	2.246733
42	1	0	0.212591	-5.568737	-1.376232
43	1	0	0.128701	-6.134704	1.894127
44	1	0	-3.907311	3.069764	-1.463338
45	1	0	-4.060058	2.106245	1.70571
46	1	0	7.207534	-1.218116	0.607748
47	1	0	7.482726	-1.733756	3.894235
48	1	0	-3.196514	-8.391799	-0.629226
49	1	0	-4.596238	-6.455551	1.673911
50	1	0	9.904619	2.418387	4.151073
51	1	0	9.513256	2.614472	0.844749
52	1	0	7.822153	6.57127	4.731894
53	1	0	10.447359	6.877486	2.714912
54	1	0	-6.987662	-3.525162	2.28862
55	1	0	-7.010735	-0.219473	1.967203
56	1	0	-9.185762	-2.048513	0.264671
57	1	0	4.430535	-0.64568	-2.767561
58	1	0	3.724476	-3.825764	-2.129027
59	1	0	1.484043	-1.866117	-3.658248
60	1	0	-1.258118	-0.015373	4.332642
61	1	0	-3.712983	-2.243938	3.93169
62	1	0	-0.719695	-3.264838	4.892697
63	1	0	-9.166901	-3.352168	-6.432195
64	1	0	-5.272939	-9.450778	-4.915944
65	1	0	-7.722085	-8.377241	-6.923716
66	1	0	-4.725909	-6.874611	-6.999442
67	1	0	-10.680065	-7.712096	-3.326404
68	1	0	-8.338327	-8.949083	-1.282606
69	1	0	-9.775821	-6.00291	-0.602621
70	1	0	8.318671	5.054952	-2.38808
71	1	0	9.866516	7.960523	-1.845942
72	1	0	6.817447	7.915211	-3.22825

73	1	0	5.242638	10.97192	0.032445
74	1	0	5.666557	10.343016	3.297872
75	1	0	8.313894	11.052491	1.368306
76	1	0	-8.607739	1.242832	-6.216288
77	1	0	-5.682603	2.270358	-4.971208
78	1	0	-7.934334	2.756586	-1.13617

Table S3. Gibbs free energies[a] and equilibrium populations[b] of low-energy conformers of compound **2**.

Conformers	ΔG(a.u.)	P(%)/100	G(a.u.)
COMPOUND 2000001_tddft_	0.00092	9.86	-1471.098817
COMPOUND 2000002_tddft_	0.00043	16.54	-1471.099305
COMPOUND 2000003_tddft_	0.00172	4.23	-1471.098017
COMPOUND 2000004_tddft_	0.00224	2.42	-1471.097492
COMPOUND 2000005_tddft_	0.00211	2.79	-1471.097624
COMPOUND 2000006_tddft_	0.00041	16.92	-1471.099327
COMPOUND 2000007_tddft_	0.00000	26.11	-1471.099736
COMPOUND 2000008_tddft_	0.00032	18.52	-1471.099412
COMPOUND 2000009_tddft_	0.00217	2.61	-1471.097562

[a]wB97M-V/def2-TZVP, in a.u.

[b]From ΔG values at 298.15K.

Table S4. Cartesian coordinates for the low-energy reoptimized random reseach conformers of compound **2** at B3LYP-D3(BJ)/6-31G* level of theory in methanol.

COMPOUND 2000001_en_		Standard Orientation (A.U.)			
Center number	Atomic number	Atomic Type	X	Y	Z
0	6	0	1.684864	1.943418	1.367015
1	6	0	-5.318281	-2.102638	-0.746919
2	6	0	1.964147	-0.828734	0.549242
3	6	0	4.134853	3.417178	1.51989
4	6	0	-0.588226	-2.312039	1.084201
5	6	0	6.179537	2.144823	3.190416
6	6	0	-2.767385	-0.723175	-0.133745
7	6	0	-4.875866	-4.8375	-1.847719
8	6	0	5.334309	1.702728	5.903378
9	6	0	4.029509	6.294246	1.922774
10	8	0	7.350614	1.146786	7.416591
11	6	0	-0.568355	3.019854	1.70732
12	6	0	4.197776	-2.071224	1.999323
13	6	0	-0.52984	-5.00862	-0.061536
14	6	0	-3.056891	1.71745	1.379699
15	6	0	6.631816	-0.511067	2.059577
16	6	0	-2.094041	-5.289156	-2.459605
17	6	0	8.643703	3.7511	3.148206
18	8	0	3.203152	1.688052	6.723579
19	6	0	6.551063	7.462998	0.920701
20	6	0	8.731244	5.529033	0.880745
21	6	0	-7.143068	-2.199936	1.524261
22	6	0	2.654176	-0.896427	-2.293712
23	6	0	-0.964295	-2.584975	3.961772
24	6	0	6.205797	8.627678	-1.698782
25	6	0	-6.745553	-0.825047	-2.926195
26	6	0	-7.510751	-2.483799	-4.664521
27	6	0	-6.691745	-5.154367	-4.167157
28	6	0	-5.503756	-6.329006	-6.542715
29	6	0	3.479932	7.166479	4.627957
30	6	0	-7.419827	1.922088	-3.106999
31	8	0	-5.332307	3.480573	-3.88918
32	6	0	-8.959519	-6.790506	-3.381972
33	8	0	-10.763908	-6.815369	-5.393651
34	1	0	4.930251	3.226587	-0.395076
35	1	0	-2.073559	-0.135131	-1.992315
36	1	0	-5.39052	-6.228928	-0.387402
37	1	0	2.50184	7.009191	0.712911

38	1	0	6.651146	0.794701	9.074349
39	1	0	-0.66541	4.990024	2.281033
40	1	0	3.643487	-2.427361	3.957203
41	1	0	4.613918	-3.919352	1.158159
42	1	0	1.417127	-5.629085	-0.392184
43	1	0	-1.300617	-6.339006	1.334597
44	1	0	-4.341068	3.021701	0.421808
45	1	0	-3.885187	1.343386	3.250138
46	1	0	7.422843	-0.250242	0.15988
47	1	0	8.066868	-1.511513	3.171982
48	1	0	-1.841857	-7.198076	-3.224392
49	1	0	-1.447735	-3.974431	-3.925447
50	1	0	8.778359	4.887153	4.874124
51	1	0	10.285715	2.487786	3.168136
52	1	0	7.097492	8.976484	2.23653
53	1	0	10.540593	6.541076	0.861859
54	1	0	8.683633	4.438793	-0.888142
55	1	0	-8.928821	-3.079046	0.963945
56	1	0	-6.366897	-3.299481	3.091778
57	1	0	-7.587499	-0.309635	2.234708
58	1	0	1.260957	0.085478	-3.459191
59	1	0	4.470891	0.020343	-2.646526
60	1	0	2.829661	-2.843783	-2.969701
61	1	0	-2.899392	-3.16915	4.379223
62	1	0	0.264302	-4.066039	4.719109
63	1	0	-0.564111	-0.840524	4.991502
64	1	0	7.988485	9.392779	-2.426406
65	1	0	4.813584	10.158882	-1.663628
66	1	0	5.537964	7.197493	-3.045035
67	1	0	-8.679306	-2.00314	-6.28612
68	1	0	-4.867311	-8.267757	-6.190842
69	1	0	-6.912171	-6.393781	-8.05284
70	1	0	-3.893733	-5.219885	-7.208518
71	1	0	3.225863	9.22236	4.652979
72	1	0	1.791834	6.281596	5.419406
73	1	0	5.051026	6.736759	5.906357
74	1	0	-8.030027	2.684776	-1.280858
75	1	0	-9.007516	2.140182	-4.433251
76	1	0	-4.627018	2.694944	-5.374899
77	1	0	-9.807509	-6.018508	-1.648316
78	1	0	-8.278537	-8.719913	-2.971781
79	1	0	-12.189975	-7.813635	-4.868737

COMPOUND 2000002_en_		Standard Orientation (A.U.)			
Center number	Atomic number	Atomic Type	X	Y	Z
0	6	0	1.639063	1.800648	1.277312
1	6	0	-5.383097	-2.345556	-0.584783
2	6	0	1.807813	-1.082001	0.947181
3	6	0	4.158383	3.153075	1.423904
4	6	0	-0.873761	-2.308538	1.496004
5	6	0	5.975638	2.067555	3.450463
6	6	0	-2.870044	-0.858951	-0.118164
7	6	0	-4.719886	-4.752669	-2.18987
8	6	0	4.893929	2.135612	6.113946
9	6	0	4.210307	6.059495	1.335816
10	8	0	6.729168	1.655961	7.861991
11	6	0	-0.557572	3.03703	1.22271
12	6	0	3.828054	-2.176288	2.782649
13	6	0	-0.983278	-5.152372	0.78685
14	6	0	-3.098656	1.850859	0.829652
15	6	0	6.34794	-0.756893	2.807251
16	6	0	-1.979689	-5.641143	-1.882805
17	6	0	8.533537	3.521013	3.374496
18	8	0	2.719053	2.448353	6.736494
19	6	0	6.881516	6.912978	0.402282
20	6	0	8.919539	4.882445	0.863651
21	6	0	-6.891259	-3.087196	1.792098
22	6	0	2.752082	-1.654232	-1.759641
23	6	0	-1.431587	-2.028777	4.350267
24	6	0	6.836023	7.662151	-2.385962
25	6	0	-7.164911	-0.912014	-2.346373
26	6	0	-7.200891	-1.887835	-4.671011
27	6	0	-5.576223	-4.205596	-4.972102
28	6	0	-3.461465	-3.732065	-6.902722
29	6	0	3.483201	7.416896	3.787454
30	6	0	-8.887229	1.164988	-1.454989
31	8	0	-10.106723	2.490986	-3.456707
32	6	0	-7.205854	-6.400833	-5.988359
33	8	0	-9.351963	-6.94392	-4.461823
34	1	0	5.086805	2.608312	-0.357349
35	1	0	-2.077079	-0.69162	-2.028522
36	1	0	-5.961134	-6.26126	-1.494808
37	1	0	2.845357	6.628706	-0.121257
38	1	0	5.885125	1.643547	9.48961
39	1	0	-0.568419	5.076525	1.470212

40	1	0	3.095809	-2.170448	4.714136
41	1	0	4.180959	-4.159339	2.294531
42	1	0	0.879017	-6.030426	1.02296
43	1	0	-2.219557	-6.124978	2.139092
44	1	0	-4.11732	2.99946	-0.565103
45	1	0	-4.209727	1.96394	2.58781
46	1	0	7.308594	-0.860637	0.971603
47	1	0	7.617028	-1.628891	4.194485
48	1	0	-1.857618	-7.66686	-2.314864
49	1	0	-0.754365	-4.695641	-3.243981
50	1	0	8.592004	4.927182	4.893428
51	1	0	10.083204	2.200744	3.75747
52	1	0	7.418777	8.584879	1.513915
53	1	0	10.790308	5.775212	0.836482
54	1	0	8.929731	3.515085	-0.701153
55	1	0	-7.301652	-1.462737	3.010341
56	1	0	-8.699367	-3.910716	1.203625
57	1	0	-5.921798	-4.513737	2.922809
58	1	0	4.59352	-0.78472	-2.107637
59	1	0	2.996256	-3.688701	-2.040906
60	1	0	1.474055	-0.924242	-3.208216
61	1	0	-3.447787	-2.152171	4.743434
62	1	0	-0.526391	-3.550222	5.421127
63	1	0	-0.751813	-0.228033	5.098362
64	1	0	8.721352	8.201954	-3.053919
65	1	0	5.555927	9.255023	-2.716131
66	1	0	6.185529	6.07534	-3.55363
67	1	0	-8.361918	-1.167843	-6.210351
68	1	0	-2.309173	-5.424568	-7.216854
69	1	0	-4.288654	-3.182736	-8.720491
70	1	0	-2.215164	-2.194618	-6.302861
71	1	0	4.906039	7.145845	5.266774
72	1	0	3.359081	9.454829	3.435745
73	1	0	1.681424	6.765942	4.554305
74	1	0	-7.908774	2.477088	-0.176321
75	1	0	-10.420804	0.322521	-0.338005
76	1	0	-8.801454	3.190972	-4.518997
77	1	0	-6.07732	-8.140546	-6.036193
78	1	0	-7.785413	-5.967533	-7.945023
79	1	0	-10.198564	-5.355906	-4.145309

COMPOUND 2000003_en_		Standard Orientation (A.U.)			
Center number	Atomic number	Atomic Type	X	Y	Z
0	6	0	1.591818	1.675858	1.46153
1	6	0	-5.44248	-2.129397	-0.882391
2	6	0	1.766846	-1.132271	0.726655
3	6	0	4.109656	3.017778	1.714075
4	6	0	-0.896076	-2.443849	1.150404
5	6	0	5.986637	1.690035	3.531297
6	6	0	-2.921109	-0.766268	-0.196622
7	6	0	-4.873191	-4.489785	-2.609969
8	6	0	4.976945	1.389779	6.207302
9	6	0	4.147213	5.908879	2.019179
10	8	0	6.870399	0.73679	7.835543
11	6	0	-0.61373	2.880012	1.677721
12	6	0	3.835382	-2.446092	2.352448
13	6	0	-1.000855	-5.160283	0.056394
14	6	0	-3.15991	1.728464	1.226471
15	6	0	6.349744	-1.02372	2.5105
16	6	0	-2.09524	-5.282152	-2.610178
17	6	0	8.536678	3.156848	3.57915
18	8	0	2.813526	1.559203	6.917086
19	6	0	6.780004	6.898002	1.11588
20	6	0	8.848584	4.850399	1.267189
21	6	0	-7.044372	-2.965337	1.404752
22	6	0	2.652453	-1.325186	-2.052589
23	6	0	-1.399313	-2.603536	4.023282
24	6	0	6.643261	7.971221	-1.561429
25	6	0	-7.17454	-0.540555	-2.56797
26	6	0	-7.562878	-1.607658	-4.815937
27	6	0	-6.02873	-3.947737	-5.280228
28	6	0	-4.097707	-3.427116	-7.391284
29	6	0	3.509334	6.912099	4.659371
30	6	0	-8.517251	1.850333	-1.810141
31	8	0	-7.255919	4.098114	-2.692121
32	6	0	-7.694475	-6.146092	-6.162912
33	8	0	-9.698233	-6.510961	-4.390269
34	1	0	4.999884	2.721802	-0.144673
35	1	0	-2.130023	-0.261716	-2.048926
36	1	0	-5.961442	-6.06292	-1.812014
37	1	0	2.72698	6.661664	0.705703
38	1	0	6.067922	0.488775	9.465239
39	1	0	-0.623956	4.862326	2.217473

40	1	0	3.148996	-2.702957	4.283276
41	1	0	4.192033	-4.342176	1.594587
42	1	0	0.875409	-6.038604	0.111553
43	1	0	-2.191385	-6.325918	1.295432
44	1	0	-4.291515	3.074234	0.137002
45	1	0	-4.140604	1.476976	3.045598
46	1	0	7.265631	-0.875687	0.654898
47	1	0	7.65494	-2.063549	3.739912
48	1	0	-1.928552	-7.213291	-3.354726
49	1	0	-0.985881	-4.082211	-3.868972
50	1	0	8.643832	4.339891	5.275232
51	1	0	10.100672	1.805971	3.725915
52	1	0	7.338299	8.433808	2.400498
53	1	0	10.709038	5.763592	1.322658
54	1	0	8.842884	3.702169	-0.465479
55	1	0	-7.358051	-1.42888	2.755417
56	1	0	-8.888546	-3.619613	0.727164
57	1	0	-6.169318	-4.540806	2.412678
58	1	0	1.348117	-0.400345	-3.360101
59	1	0	4.488909	-0.417968	-2.313326
60	1	0	2.880699	-3.30193	-2.619486
61	1	0	-3.402343	-2.873659	4.414483
62	1	0	-0.412159	-4.228791	4.838945
63	1	0	-0.772682	-0.908753	5.022159
64	1	0	5.34852	9.58311	-1.6636
65	1	0	5.961954	6.527755	-2.886835
66	1	0	8.505514	8.598277	-2.218445
67	1	0	-8.868175	-0.878489	-6.23417
68	1	0	-2.932054	-5.08772	-7.808138
69	1	0	-5.083961	-2.885255	-9.130609
70	1	0	-2.848154	-1.863804	-6.874957
71	1	0	3.381408	8.978963	4.598732
72	1	0	1.73066	6.161228	5.388143
73	1	0	4.980003	6.437687	6.037367
74	1	0	-8.641411	2.041924	0.245689
75	1	0	-10.456794	1.805687	-2.559458
76	1	0	-6.664711	3.730528	-4.376523
77	1	0	-6.517574	-7.860578	-6.32469
78	1	0	-8.45885	-5.711538	-8.053392
79	1	0	-10.637045	-7.987081	-4.887623

COMPOUND 2000004_en_		Standard Orientation (A.U.)			
Center number	Atomic number	Atomic Type	X	Y	Z
0	6	0	1.493466	1.999175	1.665155
1	6	0	-5.149985	-2.093739	-1.289108
2	6	0	1.949448	-0.698078	0.688525
3	6	0	3.867238	3.55167	2.057298
4	6	0	-0.56763	-2.317653	0.893928
5	6	0	5.89857	2.287132	3.758304
6	6	0	-2.710823	-0.702267	-0.350167
7	6	0	-4.554856	-4.761622	-2.470431
8	6	0	5.168809	1.763956	6.497334
9	6	0	3.618346	6.395119	2.606447
10	8	0	2.669591	1.359821	6.900035
11	6	0	-0.823798	2.959425	1.936365
12	6	0	4.103703	-1.948697	2.245481
13	6	0	-0.300496	-4.903469	-0.461177
14	6	0	-3.221484	1.574776	1.351229
15	6	0	6.472924	-0.324146	2.571983
16	6	0	-1.727749	-5.073506	-2.951309
17	6	0	8.322501	3.955254	3.799962
18	8	0	6.687549	1.587396	8.201509
19	6	0	6.130898	7.729387	1.800853
20	6	0	8.371706	5.876342	1.652824
21	6	0	-7.189443	-2.33046	0.78059
22	6	0	2.873696	-0.544099	-2.087382
23	6	0	-1.169377	-2.862695	3.692679
24	6	0	5.822213	9.126933	-0.707007
25	6	0	-6.278185	-0.71018	-3.580212
26	6	0	-6.862468	-2.303234	-5.447077
27	6	0	-6.211447	-5.012839	-4.91342
28	6	0	-4.892796	-6.238231	-7.189994
29	6	0	2.906629	7.072092	5.329574
30	6	0	-6.736721	2.076373	-3.776806
31	8	0	-8.485843	3.024251	-1.929016
32	6	0	-8.673043	-6.505885	-4.407499
33	8	0	-8.268033	-9.129761	-3.939836
34	1	0	4.763084	3.495829	0.180065
35	1	0	-1.883686	0.072673	-2.085446
36	1	0	-5.086465	-6.226157	-1.086564
37	1	0	2.11781	7.116293	1.366442
38	1	0	2.516908	0.967571	8.687701
39	1	0	-1.045877	4.877753	2.638988

40	1	0	3.409543	-2.425127	4.12946
41	1	0	4.642892	-3.73729	1.346053
42	1	0	1.691981	-5.391274	-0.735012
43	1	0	-1.057255	-6.386941	0.779917
44	1	0	-4.581979	2.872475	0.489868
45	1	0	-4.124631	0.979685	3.127025
46	1	0	7.409411	0.02819	0.754406
47	1	0	7.845871	-1.328297	3.758626
48	1	0	-1.36089	-6.914792	-3.825546
49	1	0	-1.067029	-3.636496	-4.293102
50	1	0	8.43166	4.967099	5.601297
51	1	0	9.994385	2.733482	3.741495
52	1	0	6.588823	9.124825	3.271283
53	1	0	10.1455	6.947019	1.72623
54	1	0	8.379821	4.912056	-0.188781
55	1	0	-8.902188	-3.182073	-0.002396
56	1	0	-6.552982	-3.53527	2.335244
57	1	0	-7.713238	-0.488768	1.550627
58	1	0	1.541471	0.46022	-3.304563
59	1	0	4.671745	0.461351	-2.221829
60	1	0	3.189815	-2.429625	-2.875448
61	1	0	-3.102934	-3.562917	3.884204
62	1	0	0.067704	-4.345608	4.431036
63	1	0	-0.948859	-1.19424	4.887232
64	1	0	5.255049	7.808573	-2.205243
65	1	0	7.596105	10.020682	-1.295683
66	1	0	4.371918	10.59848	-0.577958
67	1	0	-7.736821	-1.727241	-7.219754
68	1	0	-4.390798	-8.204089	-6.799178
69	1	0	-6.186921	-6.241507	-8.807659
70	1	0	-3.194203	-5.201598	-7.742206
71	1	0	1.192704	6.106955	5.957036
72	1	0	4.4207	6.587355	6.657659
73	1	0	2.601144	9.114686	5.484422
74	1	0	-7.397865	2.513446	-5.700739
75	1	0	-4.988666	3.147896	-3.475937
76	1	0	-9.998833	2.016526	-2.064102
77	1	0	-9.871926	-6.424708	-6.09688
78	1	0	-9.731323	-5.630908	-2.849297
79	1	0	-7.40877	-9.287849	-2.341861

COMPOUND 2000005_en_		Standard Orientation (A.U.)			
Center number	Atomic number	Atomic Type	X	Y	Z
0	6	0	1.611125	1.990309	1.450488
1	6	0	-5.26797	-2.080172	-0.987678
2	6	0	1.960371	-0.736575	0.518632
3	6	0	4.027942	3.513075	1.670447
4	6	0	-0.569438	-2.299693	0.922227
5	6	0	6.140055	2.247408	3.268795
6	6	0	-2.756035	-0.688147	-0.250953
7	6	0	-4.757905	-4.76383	-2.176962
8	6	0	5.575089	1.78822	6.057344
9	6	0	3.855275	6.370558	2.175808
10	8	0	3.09843	1.458718	6.629011
11	6	0	-0.669148	3.000181	1.820511
12	6	0	4.187706	-1.985789	1.971193
13	6	0	-0.424388	-4.929467	-0.362941
14	6	0	-3.123972	1.663644	1.381549
15	6	0	6.597046	-0.394601	2.100528
16	6	0	-1.962802	-5.127558	-2.78442
17	6	0	8.587656	3.875855	3.129259
18	8	0	7.197164	1.601422	7.663282
19	6	0	6.335522	7.646147	1.193633
20	6	0	8.533828	5.753009	0.943622
21	6	0	-7.151743	-2.284437	1.228314
22	6	0	2.710209	-0.668284	-2.310817
23	6	0	-1.006041	-2.741452	3.768945
24	6	0	5.897039	9.005634	-1.315656
25	6	0	-6.649535	-0.734872	-3.15637
26	6	0	-7.35032	-2.34154	-4.969828
27	6	0	-6.539184	-5.024325	-4.529786
28	6	0	-5.316973	-6.141501	-6.916617
29	6	0	3.321284	7.117134	4.920565
30	6	0	-7.329724	2.015234	-3.268435
31	8	0	-5.221964	3.612112	-3.896478
32	6	0	-8.880701	-6.618195	-3.89236
33	8	0	-8.089436	-9.162105	-3.430543
34	1	0	4.81096	3.411629	-0.254977
35	1	0	-2.035697	0.014701	-2.058161
36	1	0	-5.260328	-6.229312	-0.795191
37	1	0	2.294625	7.091614	1.012655
38	1	0	3.052941	1.097592	8.429025
39	1	0	-0.817408	4.941658	2.477354

40	1	0	3.60939	-2.40149	3.908215
41	1	0	4.641975	-3.805662	1.088111
42	1	0	1.542392	-5.473651	-0.708759
43	1	0	-1.163813	-6.357248	0.950942
44	1	0	-4.417054	2.989595	0.465166
45	1	0	-3.986213	1.179185	3.211042
46	1	0	7.4083	-0.093831	0.21505
47	1	0	8.03397	-1.395924	3.211612
48	1	0	-1.672216	-6.996182	-3.628535
49	1	0	-1.329267	-3.734159	-4.183368
50	1	0	8.817972	4.92362	4.898302
51	1	0	10.235282	2.627102	2.998287
52	1	0	6.907856	9.057813	2.607602
53	1	0	10.324617	6.796475	0.886674
54	1	0	8.41454	4.754774	-0.87592
55	1	0	-7.673017	-0.426959	1.973425
56	1	0	-8.892618	-3.204724	0.600029
57	1	0	-6.384333	-3.40046	2.78794
58	1	0	2.960125	-2.578919	-3.062499
59	1	0	1.308841	0.315401	-3.465343
60	1	0	4.502434	0.317273	-2.589877
61	1	0	-2.925592	-3.423541	4.101561
62	1	0	0.265198	-4.206654	4.484909
63	1	0	-0.709377	-1.033529	4.889094
64	1	0	5.214883	7.672728	-2.751505
65	1	0	7.646712	9.856358	-2.028096
66	1	0	4.483021	10.505654	-1.125255
67	1	0	-8.458705	-1.798676	-6.618861
68	1	0	-4.69392	-8.085777	-6.608713
69	1	0	-6.70886	-6.163296	-8.451588
70	1	0	-3.706686	-5.00897	-7.541702
71	1	0	3.064226	9.168536	5.047016
72	1	0	1.630208	6.201434	5.672273
73	1	0	4.903149	6.631293	6.167016
74	1	0	-8.024898	2.702957	-1.442885
75	1	0	-8.860591	2.276735	-4.653231
76	1	0	-4.464898	2.912395	-5.399625
77	1	0	-10.215043	-6.540273	-5.493732
78	1	0	-9.833398	-5.827609	-2.223135
79	1	0	-9.54687	-10.130619	-2.935613

COMPOUND 2000006_en_		Standard Orientation (A.U.)			
Center number	Atomic number	Atomic Type	X	Y	Z
0	6	0	1.606483	1.677056	1.518906
1	6	0	-5.47581	-2.172835	-0.705306
2	6	0	1.743209	-1.152213	0.865721
3	6	0	4.139551	2.988743	1.782241
4	6	0	-0.942853	-2.413177	1.305928
5	6	0	5.970021	1.673086	3.654511
6	6	0	-2.946971	-0.767102	-0.095885
7	6	0	-4.848854	-4.367957	-2.601776
8	6	0	4.914946	1.449114	6.319525
9	6	0	4.211188	5.885701	2.023461
10	8	0	6.766392	0.787696	7.988697
11	6	0	-0.578993	2.92785	1.645719
12	6	0	3.774687	-2.456347	2.545666
13	6	0	-1.086276	-5.153551	0.268181
14	6	0	-3.137871	1.812138	1.171832
15	6	0	6.307596	-1.067	2.69763
16	6	0	-2.119211	-5.325108	-2.426413
17	6	0	8.539898	3.105225	3.710075
18	8	0	2.744103	1.682833	6.98956
19	6	0	6.874983	6.822298	1.154095
20	6	0	8.908661	4.746995	1.369338
21	6	0	-6.957032	-3.190778	1.583028
22	6	0	2.651272	-1.42458	-1.898037
23	6	0	-1.464629	-2.470371	4.18063
24	6	0	6.801678	7.864447	-1.537846
25	6	0	-7.27015	-0.527328	-2.255326
26	6	0	-7.333723	-1.206044	-4.683047
27	6	0	-5.715337	-3.468481	-5.287869
28	6	0	-3.61009	-2.748158	-7.151651
29	6	0	3.531673	6.959162	4.625369
30	6	0	-8.96782	1.432507	-1.093995
31	8	0	-10.189503	3.012091	-2.900335
32	6	0	-7.347979	-5.514405	-6.573545
33	8	0	-9.492847	-6.2486	-5.127421
34	1	0	5.0499	2.639736	-0.056911
35	1	0	-2.171976	-0.37303	-1.980246
36	1	0	-6.109536	-5.934185	-2.094366
37	1	0	2.830351	6.626457	0.661774
38	1	0	5.936936	0.59124	9.611968
39	1	0	-0.564507	4.924892	2.127557

40	1	0	3.063712	-2.654627	4.474431
41	1	0	4.108009	-4.375364	1.838097
42	1	0	0.772067	-6.064221	0.375878
43	1	0	-2.312778	-6.269625	1.513615
44	1	0	-4.177335	3.123099	-0.054122
45	1	0	-4.207121	1.725	2.95713
46	1	0	7.24556	-0.9742	0.849574
47	1	0	7.583837	-2.096274	3.965336
48	1	0	-2.036448	-7.292927	-3.078714
49	1	0	-0.89063	-4.259055	-3.692765
50	1	0	8.638015	4.321668	5.383155
51	1	0	10.082093	1.736313	3.908807
52	1	0	7.434231	8.364636	2.430137
53	1	0	10.781696	5.633269	1.432276
54	1	0	8.908725	3.565827	-0.341042
55	1	0	-7.350452	-1.722215	2.990024
56	1	0	-8.773581	-3.936978	0.922053
57	1	0	-5.979432	-4.74259	2.526907
58	1	0	1.358768	-0.536367	-3.241997
59	1	0	4.49001	-0.52454	-2.168974
60	1	0	2.884428	-3.415915	-2.406925
61	1	0	-0.766035	-0.774235	5.128566
62	1	0	-3.476794	-2.63094	4.579809
63	1	0	-0.555609	-4.111835	5.052746
64	1	0	6.126357	6.414521	-2.859261
65	1	0	8.683525	8.459684	-2.167514
66	1	0	5.531059	9.492327	-1.68272
67	1	0	-8.506114	-0.296399	-6.109365
68	1	0	-2.468413	-4.386323	-7.704192
69	1	0	-4.446412	-1.95393	-8.872344
70	1	0	-2.352632	-1.31037	-6.3596
71	1	0	4.966072	6.49609	6.044505
72	1	0	3.435831	9.025655	4.514517
73	1	0	1.728154	6.250335	5.33547
74	1	0	-7.969555	2.563636	0.334202
75	1	0	-10.500602	0.465477	-0.081311
76	1	0	-8.886021	3.806612	-3.896375
77	1	0	-6.220789	-7.234286	-6.843328
78	1	0	-7.927625	-4.833564	-8.45827
79	1	0	-10.351848	-4.716002	-4.626446

COMPOUND 2000007_en_		Standard Orientation (A.U.)			
Center number	Atomic number	Atomic Type	X	Y	Z
0	6	0	1.723071	1.700208	1.448949
1	6	0	-5.28177	-2.011478	-1.153684
2	6	0	1.854727	-1.142666	0.855502
3	6	0	4.258985	2.981981	1.807739
4	6	0	-0.869521	-2.358472	1.162475
5	6	0	5.976093	1.68379	3.796461
6	6	0	-2.754749	-0.697038	-0.385243
7	6	0	-4.687214	-4.462533	-2.74091
8	6	0	4.79017	1.540462	6.412661
9	6	0	4.36408	5.88201	1.987871
10	8	0	6.550451	0.895532	8.184828
11	6	0	-0.4495	2.979866	1.458097
12	6	0	3.768528	-2.434815	2.674807
13	6	0	-0.9892	-5.114476	0.172583
14	6	0	-2.994611	1.87507	0.892714
15	6	0	6.314619	-1.084121	2.923932
16	6	0	-1.935472	-5.320933	-2.544471
17	6	0	8.562157	3.079185	3.94285
18	8	0	2.593871	1.819249	6.972402
19	6	0	7.08104	6.759568	1.227722
20	6	0	9.068058	4.658438	1.584675
21	6	0	-7.043017	-2.679656	1.068662
22	6	0	2.907966	-1.489151	-1.84823
23	6	0	-1.5634	-2.376562	3.998937
24	6	0	7.152719	7.746533	-1.484803
25	6	0	-6.778434	-0.41198	-3.040852
26	6	0	-6.944262	-1.487681	-5.310952
27	6	0	-5.671496	-4.016482	-5.495953
28	6	0	-3.65803	-3.992315	-7.585443
29	6	0	3.578413	7.024091	4.529765
30	6	0	-8.140259	1.984857	-2.372672
31	8	0	-10.436653	2.103701	-3.806962
32	6	0	-7.654951	-6.034318	-6.206095
33	8	0	-9.731305	-6.133555	-4.50126
34	1	0	5.249345	2.578577	0.021699
35	1	0	-1.849283	-0.313642	-2.212957
36	1	0	-5.858198	-5.969119	-1.92985
37	1	0	3.061718	6.610996	0.544767
38	1	0	5.640298	0.750426	9.769833
39	1	0	-0.431932	4.983866	1.909429

40	1	0	2.95082	-2.564691	4.566936
41	1	0	4.10233	-4.379245	2.039804
42	1	0	0.85561	-6.037515	0.368606
43	1	0	-2.278467	-6.198073	1.386912
44	1	0	-4.004692	3.203589	-0.333113
45	1	0	-4.1116	1.760511	2.645346
46	1	0	7.345749	-1.051344	1.124413
47	1	0	7.507983	-2.102138	4.278514
48	1	0	-1.785522	-7.287191	-3.191587
49	1	0	-0.721809	-4.213844	-3.793817
50	1	0	8.596187	4.335472	5.588706
51	1	0	10.071644	1.694196	4.251114
52	1	0	7.604045	8.31951	2.497873
53	1	0	10.950362	5.517193	1.716151
54	1	0	9.128735	3.43861	-0.097065
55	1	0	-7.335971	-1.094491	2.365663
56	1	0	-8.889395	-3.249546	0.32393
57	1	0	-6.314442	-4.274528	2.158214
58	1	0	1.712179	-0.597949	-3.276588
59	1	0	4.780969	-0.640292	-2.033362
60	1	0	3.116033	-3.495645	-2.306241
61	1	0	-3.595684	-2.573875	4.263434
62	1	0	-0.685664	-3.992345	4.946969
63	1	0	-0.948619	-0.658817	4.966809
64	1	0	6.522094	6.278762	-2.808527
65	1	0	9.070526	8.305344	-2.034042
66	1	0	5.911957	9.386369	-1.722073
67	1	0	-7.987464	-0.678933	-6.888081
68	1	0	-2.683375	-5.813203	-7.740267
69	1	0	-4.550489	-3.593216	-9.411436
70	1	0	-2.242992	-2.520698	-7.258448
71	1	0	4.944635	6.583566	6.021536
72	1	0	3.507419	9.088135	4.366158
73	1	0	1.73915	6.345524	5.173755
74	1	0	-6.95006	3.638501	-2.809584
75	1	0	-8.539814	2.031087	-0.331496
76	1	0	-11.207761	3.721108	-3.485322
77	1	0	-6.781054	-7.91546	-6.163832
78	1	0	-8.31545	-5.683406	-8.153393
79	1	0	-10.356194	-4.424	-4.323092

COMPOUND 2000008_en_		Standard Orientation (A.U.)			
Center number	Atomic number	Atomic Type	X	Y	Z
0	6	0	1.899793	1.624168	1.276976
1	6	0	-5.444697	-1.812516	-0.680254
2	6	0	1.865037	-1.222581	0.687188
3	6	0	4.505999	2.801696	1.40414
4	6	0	-0.868956	-2.326667	1.240388
5	6	0	6.335185	1.442382	3.245904
6	6	0	-2.813409	-0.594499	-0.149585
7	6	0	-5.087273	-4.281684	-2.312622
8	6	0	5.373214	1.351304	5.954298
9	6	0	4.740784	5.694931	1.558361
10	8	0	7.256252	0.662241	7.577128
11	6	0	-0.211786	2.989277	1.464414
12	6	0	3.881186	-2.591642	2.33394
13	6	0	-1.188486	-5.081125	0.291841
14	6	0	-2.838809	1.988037	1.126471
15	6	0	6.488436	-1.339389	2.363873
16	6	0	-2.372197	-5.2693	-2.331404
17	6	0	8.979129	2.730911	3.156734
18	8	0	3.240738	1.69833	6.694222
19	6	0	7.414781	6.464842	0.563863
20	6	0	9.339207	4.28616	0.75589
21	6	0	-7.005822	-2.435899	1.703403
22	6	0	2.65933	-1.615917	-2.099613
23	6	0	-1.303172	-2.302419	4.128616
24	6	0	7.290573	7.444559	-2.149513
25	6	0	-7.077594	-0.163246	-2.408191
26	6	0	-7.524128	-1.249948	-4.637817
27	6	0	-6.266797	-3.770377	-4.978442
28	6	0	-4.377807	-3.628281	-7.182876
29	6	0	4.22044	6.869655	4.154009
30	6	0	-8.288606	2.286269	-1.607527
31	8	0	-10.616432	2.784623	-2.877516
32	6	0	-8.287757	-5.773119	-5.589844
33	8	0	-7.312626	-8.286894	-5.709656
34	1	0	5.319904	2.35008	-0.456994
35	1	0	-2.053856	-0.258318	-2.05138
36	1	0	-6.243704	-5.7579	-1.42279
37	1	0	3.347658	6.472319	0.229978
38	1	0	6.478864	0.551861	9.233946
39	1	0	-0.07774	4.992902	1.896871

40	1	0	3.227312	-2.697469	4.289967
41	1	0	4.088318	-4.545413	1.673588
42	1	0	0.628479	-6.075044	0.343827
43	1	0	-2.41037	-6.100585	1.625249
44	1	0	-3.895347	3.354191	-0.013346
45	1	0	-3.804932	1.9229	2.96866
46	1	0	7.370781	-1.35067	0.486287
47	1	0	7.749324	-2.399942	3.621391
48	1	0	-2.385084	-7.241006	-2.973032
49	1	0	-1.2241	-4.222222	-3.690241
50	1	0	9.212874	3.984616	4.788145
51	1	0	10.45203	1.28466	3.332634
52	1	0	8.105198	8.003683	1.778383
53	1	0	11.259385	5.066444	0.715425
54	1	0	9.198961	3.061583	-0.917476
55	1	0	-6.233707	-4.067082	2.705387
56	1	0	-7.114468	-0.854743	3.032473
57	1	0	-8.939109	-2.925871	1.139544
58	1	0	4.552719	-0.867944	-2.449759
59	1	0	2.719453	-3.628958	-2.571328
60	1	0	1.395193	-0.657263	-3.422251
61	1	0	-3.306854	-2.416323	4.589775
62	1	0	-0.406146	-3.947109	5.006315
63	1	0	-0.536044	-0.605941	5.022046
64	1	0	6.487506	6.00121	-3.404954
65	1	0	9.174659	7.922844	-2.866322
66	1	0	6.102124	9.133768	-2.287813
67	1	0	-8.685884	-0.404411	-6.109641
68	1	0	-3.326738	-5.395866	-7.434545
69	1	0	-5.389069	-3.214581	-8.944529
70	1	0	-2.996454	-2.116371	-6.895083
71	1	0	2.415296	6.267234	4.951599
72	1	0	5.687556	6.373842	5.528146
73	1	0	4.219372	8.93471	3.989076
74	1	0	-7.076698	3.901235	-2.074062
75	1	0	-8.549738	2.310715	0.458492
76	1	0	-11.628333	1.273259	-2.748453
77	1	0	-9.263874	-5.270506	-7.363096
78	1	0	-9.700194	-5.79407	-4.07501
79	1	0	-6.260874	-8.414355	-7.189691

COMPOUND 2000009_en_		Standard Orientation (A.U.)			
Center number	Atomic number	Atomic Type	X	Y	Z
0	6	0	1.712304	1.842629	1.631687
1	6	0	-5.223173	-1.862188	-1.219349
2	6	0	1.984754	-0.868071	0.628379
3	6	0	4.178383	3.235971	2.044701
4	6	0	-0.626253	-2.321485	0.874627
5	6	0	6.081004	1.79694	3.748028
6	6	0	-2.696043	-0.589982	-0.336568
7	6	0	-4.749173	-4.496634	-2.540745
8	6	0	5.04854	1.202594	6.366212
9	6	0	4.112121	6.079695	2.641447
10	8	0	6.945791	0.502182	7.967306
11	6	0	-0.533048	2.950377	1.919619
12	6	0	4.079558	-2.270439	2.137619
13	6	0	-0.548929	-4.912893	-0.490925
14	6	0	-3.021582	1.721503	1.367278
15	6	0	6.542101	-0.791722	2.473304
16	6	0	-1.939127	-4.943364	-3.010075
17	6	0	8.58095	3.331113	3.965901
18	8	0	2.866107	1.186688	7.039056
19	6	0	6.721962	7.246561	1.891644
20	6	0	8.850509	5.25707	1.838974
21	6	0	-7.17227	-2.19607	0.928821
22	6	0	2.875657	-0.744711	-2.15787
23	6	0	-1.204034	-2.817664	3.69005
24	6	0	6.575236	8.61653	-0.645793
25	6	0	-6.431957	-0.348075	-3.389943
26	6	0	-7.085423	-1.826757	-5.322057
27	6	0	-6.425046	-4.561475	-4.974478
28	6	0	-5.133642	-5.633255	-7.341051
29	6	0	3.401328	6.775348	5.360892
30	6	0	-6.916529	2.445548	-3.417606
31	8	0	-8.748913	3.293406	-1.605475
32	6	0	-8.823613	-6.143483	-4.436504
33	8	0	-10.642435	-6.08902	-6.423531
34	1	0	5.08525	3.153485	0.173191
35	1	0	-1.875537	0.135467	-2.096515
36	1	0	-5.371601	-6.005735	-1.249759
37	1	0	2.686165	6.918732	1.387314
38	1	0	6.131639	0.059785	9.54953
39	1	0	-0.625241	4.870778	2.641663

40	1	0	3.376886	-2.738354	4.022654
41	1	0	4.507416	-4.071207	1.204314
42	1	0	1.402924	-5.56111	-0.724078
43	1	0	-1.459748	-6.328867	0.724584
44	1	0	-4.260312	3.136876	0.500567
45	1	0	-3.933127	1.208419	3.164112
46	1	0	7.475631	-0.429176	0.656506
47	1	0	7.867991	-1.906461	3.611762
48	1	0	-1.661961	-6.781597	-3.925514
49	1	0	-1.174569	-3.514246	-4.301886
50	1	0	8.637462	4.34163	5.77225
51	1	0	10.186409	2.022092	3.994648
52	1	0	7.218162	8.638072	3.353454
53	1	0	10.682602	6.214134	1.996785
54	1	0	8.879981	4.294901	-0.002686
55	1	0	-6.624758	-3.727911	2.204639
56	1	0	-7.407141	-0.497922	2.084722
57	1	0	-9.02784	-2.655468	0.144489
58	1	0	1.586735	0.345437	-3.347281
59	1	0	4.727086	0.156366	-2.309663
60	1	0	3.070221	-2.640246	-2.962274
61	1	0	-3.193651	-3.311104	3.939988
62	1	0	-0.107378	-4.427201	4.384001
63	1	0	-0.77152	-1.193572	4.890484
64	1	0	5.962188	7.312824	-2.138812
65	1	0	8.41923	9.38208	-1.198482
66	1	0	5.222106	10.181332	-0.578829
67	1	0	-8.065331	-1.15882	-7.000346
68	1	0	-4.67358	-7.643002	-7.124481
69	1	0	-6.387425	-5.428573	-8.977034
70	1	0	-3.395285	-4.615433	-7.795205
71	1	0	4.878506	6.223313	6.702898
72	1	0	3.180886	8.829163	5.515891
73	1	0	1.652155	5.8706	5.976616
74	1	0	-7.70538	2.959237	-5.259251
75	1	0	-5.133444	3.503488	-3.206637
76	1	0	-8.194773	2.788852	0.053225
77	1	0	-9.793218	-5.384513	-2.775689
78	1	0	-8.25282	-8.102448	-4.002705
79	1	0	-10.027619	-7.122793	-7.788078

Table S5. Gibbs free energies[a] and equilibrium populations[b] of low-energy conformers of compound **22**.

Conformers	ΔG(a.u.)	P(%)/100	G(a.u.)
COMPOUND 22000002_tddft_	0.00139	8.04	-1540.727687
COMPOUND 22000003_tddft_	0.00020	28.34	-1540.728876
COMPOUND 22000005_tddft_	0.00019	28.58	-1540.728884
COMPOUND 22000009_tddft_	0.00000	35.04	-1540.729077

[a]wB97M-V/def2-TZVP, in a.u.

[b]From ΔG values at 298.15K.

Table S6.Cartesian coordinates for the low-energy reoptimized random reseach conformers of compound **22** at B3LYP-D3(BJ)/6-31G* level of theory in methanol.

COMPOUND 22000002_en_		Standard Orientation (A.U.)			
Center number	Atomic number	Atomic Type	X	Y	Z
0	6	0	-9.553027	-2.853556	-2.715206
1	6	0	-8.369913	-5.398256	-2.068572
2	6	0	-5.486564	-5.45483	-2.440669
3	6	0	-4.300393	-3.223353	-0.912413
4	6	0	-5.396221	-0.569682	-1.567103
5	6	0	-8.304468	-0.703689	-1.247331
6	6	0	-1.411455	-3.172307	-0.725855
7	6	0	-0.617428	-1.436004	1.432239
8	6	0	-1.503992	1.279268	1.044966
9	6	0	-4.38001	1.353701	0.453925
10	6	0	0.063501	2.728549	-0.937133
11	6	0	-0.669571	5.474585	-0.397858
12	6	0	-3.38656	5.863365	-1.323268
13	6	0	-5.259829	4.124194	0.062312
14	6	0	-0.956831	2.898877	3.431297
15	6	0	-0.539868	5.65532	2.523253
16	1	0	-2.056347	6.897291	3.213282
17	6	0	1.884395	6.83641	3.518214
18	6	0	-4.821886	0.225994	-4.299349
19	6	0	-4.502893	-8.012659	-1.424568
20	8	0	4.07917	5.489723	2.584608
21	6	0	5.439156	4.112125	4.259655

22	6	0	7.524422	2.72662	3.017234
23	8	0	4.996773	4.017558	6.512129
24	6	0	9.073144	1.234152	4.569127
25	6	0	11.044676	-0.114915	3.517935
26	6	0	11.497102	0.00144	0.895214
27	6	0	9.958104	1.476776	-0.632209
28	6	0	7.976596	2.839899	0.421697
29	8	0	13.42975	-1.320464	-0.144953
30	1	0	-4.973459	-3.61026	1.027368
31	1	0	-5.282794	0.704865	2.21603
32	6	0	-4.899083	-5.482913	-5.260632
33	8	0	-6.425886	-5.765118	-6.9412
34	8	0	-2.382359	-5.324686	-5.757983
35	8	0	12.700339	-1.635861	4.800395
36	6	0	12.37496	-1.836619	7.469522
37	1	0	-11.575714	-2.900076	-2.260978
38	1	0	-9.432684	-2.531082	-4.756268
39	1	0	-8.731599	-5.833616	-0.069034
40	1	0	-9.23649	-6.903212	-3.194355
41	1	0	-8.728265	-0.94795	0.774296
42	1	0	-9.165871	1.091414	-1.820143
43	1	0	-0.559939	-2.53902	-2.500729
44	1	0	-0.674732	-5.073993	-0.380215
45	1	0	1.448021	-1.441423	1.653297
46	1	0	-1.40896	-2.165826	3.213747
47	1	0	-0.319419	2.193259	-2.89315
48	1	0	2.077802	2.41111	-0.564413
49	1	0	0.599019	6.83671	-1.313827
50	1	0	-3.434825	5.524085	-3.368027
51	1	0	-3.97167	7.834336	-1.034956
52	1	0	-7.08886	4.182722	-0.902533
53	1	0	-5.617419	4.935443	1.937008
54	1	0	-2.470652	2.748144	4.83769
55	1	0	0.768407	2.182074	4.329015
56	1	0	2.089703	8.775263	2.818479
57	1	0	1.925837	6.838904	5.585779
58	1	0	-2.823837	0.073945	-4.786569
59	1	0	-5.867057	-0.927814	-5.656881
60	1	0	-5.414779	2.181513	-4.612719
61	1	0	-5.584082	-9.564397	-2.266492
62	1	0	-2.507478	-8.316394	-1.848649
63	1	0	-4.763908	-8.083554	0.628106
64	1	0	8.665953	1.188781	6.575804

65	1	0	10.344303	1.538745	-2.645551
66	1	0	6.783315	3.998547	-0.772679
67	1	0	14.278385	-2.217655	1.205055
68	1	0	-2.221968	-5.456886	-7.580558
69	1	0	12.580918	0.013803	8.382255
70	1	0	10.518407	-2.636135	7.931992
71	1	0	13.859982	-3.10504	8.128905

COMPOUND 22000003_en_		Standard Orientation (A.U.)			
Center number	Atomic number	Atomic Type	X	Y	Z
0	6	0	-9.29767	-3.974437	-2.947804
1	6	0	-7.783595	-5.149386	-5.097485
2	6	0	-5.906404	-3.302222	-6.330539
3	6	0	-4.191191	-2.160681	-4.21572
4	6	0	-5.661174	-0.860259	-2.019835
5	6	0	-7.565965	-2.811701	-0.952023
6	6	0	-1.936565	-0.570703	-5.090747
7	6	0	-0.05304	-0.295682	-2.927152
8	6	0	-1.196576	1.031685	-0.639293
9	6	0	-3.719387	-0.254777	0.142584
10	6	0	-1.486766	3.906435	-1.00343
11	6	0	-1.920103	4.818774	1.707731
12	6	0	-4.564905	3.986478	2.534651
13	6	0	-4.856152	1.096586	2.480449
14	6	0	0.656869	0.975634	1.639171
15	6	0	0.153223	3.401255	3.211835
16	1	0	-0.520605	2.936399	5.118329
17	6	0	2.492598	5.043454	3.487912
18	6	0	-7.151353	1.469792	-2.904424
19	6	0	-4.288517	-4.813978	-8.23956
20	8	0	4.41958	3.681951	4.877883
21	6	0	6.539777	2.983224	3.62553
22	6	0	8.293158	1.554362	5.267804
23	8	0	6.954053	3.471572	1.418171
24	6	0	10.567466	0.729471	4.180914
25	6	0	12.278429	-0.615489	5.622367
26	6	0	11.743145	-1.158746	8.174445
27	6	0	9.490243	-0.341103	9.239655
28	6	0	7.766519	1.012019	7.793757
29	8	0	13.41689	-2.47814	9.597137
30	1	0	-3.354765	-3.871787	-3.356443
31	1	0	-3.144315	-2.147969	0.795513
32	6	0	-7.364184	-1.392819	-7.920023
33	8	0	-9.591294	-1.504403	-8.431694
34	8	0	-5.860058	0.445213	-8.904008
35	8	0	14.560822	-1.531758	4.812751
36	6	0	15.234888	-1.064991	2.244585
37	1	0	-10.473633	-5.432544	-2.058435
38	1	0	-10.611152	-2.567741	-3.709355

39	1	0	-6.667351	-6.738714	-4.358158
40	1	0	-9.052886	-5.907489	-6.545667
41	1	0	-6.489308	-4.339266	-0.039368
42	1	0	-8.723978	-1.936688	0.526534
43	1	0	-2.54561	1.292599	-5.749587
44	1	0	-0.999584	-1.470189	-6.700807
45	1	0	1.626163	0.756595	-3.545547
46	1	0	0.607173	-2.185588	-2.358158
47	1	0	-3.012002	4.472017	-2.27332
48	1	0	0.286524	4.666814	-1.772482
49	1	0	-1.743242	6.879105	1.894853
50	1	0	-5.952116	4.899834	1.29374
51	1	0	-4.957725	4.660508	4.457916
52	1	0	-6.851021	0.602519	2.717291
53	1	0	-3.910588	0.333906	4.161016
54	1	0	0.448252	-0.755526	2.755734
55	1	0	2.597726	0.998727	0.910931
56	1	0	3.253113	5.608076	1.648791
57	1	0	2.080387	6.73229	4.613817
58	1	0	-5.998645	2.814175	-3.961725
59	1	0	-8.733791	0.939671	-4.121526
60	1	0	-7.967448	2.45612	-1.281626
61	1	0	-3.089678	-6.155101	-7.2145
62	1	0	-5.522396	-5.888589	-9.507498
63	1	0	-3.084745	-3.598958	-9.39208
64	1	0	10.913861	1.182051	2.213751
65	1	0	9.119181	-0.782661	11.207896
66	1	0	6.008465	1.648774	8.627359
67	1	0	14.854925	-2.87472	8.537933
68	1	0	-6.955784	1.483418	-9.946411
69	1	0	17.083265	-1.936941	1.975318
70	1	0	13.861969	-1.92507	0.950211
71	1	0	15.362215	0.970493	1.86973

COMPOUND 22000005_en_		Standard Orientation (A.U.)			
Center number	Atomic number	Atomic Type	X	Y	Z
0	6	0	-10.149997	-2.14755	-3.476707
1	6	0	-9.407177	-4.838758	-2.770028
2	6	0	-6.537734	-5.304169	-2.863732
3	6	0	-5.204183	-3.269702	-1.188964
4	6	0	-5.844726	-0.48461	-1.904145
5	6	0	-8.759219	-0.208785	-1.851114
6	6	0	-2.372102	-3.638877	-0.715249
7	6	0	-1.553188	-2.022652	1.524209
8	6	0	-1.98954	0.79053	1.076606
9	6	0	-4.758489	1.278739	0.223556
10	6	0	-0.058523	1.989339	-0.745186
11	6	0	-0.42554	4.814019	-0.246561
12	6	0	-2.961645	5.591597	-1.410172
13	6	0	-5.186278	4.146773	-0.219944
14	6	0	-1.435115	2.324516	3.518674
15	6	0	-0.537995	4.985667	2.675879
16	1	0	-1.91847	6.434979	3.230125
17	6	0	1.913866	5.81585	3.906282
18	6	0	-4.909265	0.217267	-4.560968
19	6	0	-6.031325	-7.980393	-1.80353
20	8	0	3.881493	4.016466	3.285285
21	6	0	6.183222	4.521022	4.28099
22	6	0	8.073882	2.562657	3.648013
23	8	0	6.620279	6.383353	5.55267
24	6	0	10.555249	2.962415	4.49458
25	6	0	12.40323	1.194542	3.971331
26	6	0	11.799921	-1.004982	2.594743
27	6	0	9.342205	-1.394659	1.766517
28	6	0	7.481652	0.382473	2.289886
29	8	0	13.607396	-2.746074	2.077702
30	1	0	-6.112999	-3.566583	0.669114
31	1	0	-5.907926	0.776778	1.887659
32	6	0	-5.688722	-5.408036	-5.61511
33	8	0	-7.0715	-5.478985	-7.436328
34	8	0	-3.137602	-5.593491	-5.868672
35	8	0	14.889794	1.340386	4.675104
36	6	0	15.644204	3.506158	6.092917
37	1	0	-12.193565	-1.908537	-3.219394
38	1	0	-9.786508	-1.833838	-5.489934

39	1	0	-10.021448	-5.229533	-0.823819
40	1	0	-10.362643	-6.198133	-4.003748
41	1	0	-9.392532	-0.419411	0.118668
42	1	0	-9.307774	1.697114	-2.449923
43	1	0	-1.258847	-3.157899	-2.390244
44	1	0	-1.965379	-5.625621	-0.306228
45	1	0	0.455688	-2.331026	1.95367
46	1	0	-2.618713	-2.614553	3.211492
47	1	0	-0.338022	1.511712	-2.733132
48	1	0	1.848785	1.38844	-0.206228
49	1	0	1.106872	5.97139	-1.030047
50	1	0	-2.87068	5.259265	-3.454368
51	1	0	-3.278037	7.627267	-1.154236
52	1	0	-6.890967	4.46862	-1.347058
53	1	0	-5.592738	5.007539	1.622423
54	1	0	-3.081224	2.397736	4.775198
55	1	0	0.089209	1.373148	4.554249
56	1	0	2.494965	7.691227	3.242096
57	1	0	1.729041	5.901899	5.970382
58	1	0	-2.919548	-0.234526	-4.857144
59	1	0	-5.983772	-0.770544	-6.021833
60	1	0	-5.173378	2.2383	-4.903574
61	1	0	-4.07024	-8.571571	-2.04834
62	1	0	-6.489018	-8.01915	0.215396
63	1	0	-7.242454	-9.355774	-2.766288
64	1	0	10.946593	4.673201	5.550675
65	1	0	8.920307	-3.104459	0.715287
66	1	0	5.56358	0.068581	1.646073
67	1	0	15.177162	-2.149144	2.803498
68	1	0	-2.822339	-5.745183	-7.66948
69	1	0	17.656015	3.264397	6.471343
70	1	0	15.343005	5.245519	5.005042
71	1	0	14.605079	3.618112	7.883795

COMPOUND 22000009_en_		Standard Orientation (A.U.)			
Center number	Atomic number	Atomic Type	X	Y	Z
0	6	0	-9.648898	-2.790568	-2.642802
1	6	0	-8.506618	-5.311618	-1.849084
2	6	0	-5.619968	-5.420633	-2.179555
3	6	0	-4.424251	-3.128777	-0.751995
4	6	0	-5.485097	-0.497616	-1.545388
5	6	0	-8.397892	-0.585959	-1.261557
6	6	0	-1.53601	-3.094952	-0.545477
7	6	0	-0.7413	-1.268218	1.536176
8	6	0	-1.603627	1.434046	1.022682
9	6	0	-4.473876	1.504034	0.400287
10	6	0	-0.008481	2.788362	-1.004167
11	6	0	-0.725781	5.560306	-0.585372
12	6	0	-3.430477	5.930448	-1.552133
13	6	0	-5.329528	4.261059	-0.116737
14	6	0	-1.066586	3.14834	3.344481
15	6	0	-0.620477	5.861022	2.326504
16	1	0	-2.133013	7.142102	2.950527
17	6	0	1.80496	7.063337	3.292208
18	6	0	-4.863066	0.161035	-4.30384
19	6	0	-4.680679	-7.934822	-1.021315
20	8	0	3.997134	5.676309	2.412888
21	6	0	5.352265	4.357824	4.138931
22	6	0	7.445297	2.936859	2.951039
23	8	0	4.90081	4.338062	6.391364
24	6	0	8.995674	1.505401	4.557763
25	6	0	10.977875	0.131948	3.559391
26	6	0	11.43933	0.162609	0.935779
27	6	0	9.898044	1.577231	-0.64595
28	6	0	7.906085	2.96478	0.354775
29	8	0	13.382872	-1.182563	-0.052888
30	1	0	-5.116842	-3.414636	1.198351
31	1	0	-5.397669	0.935037	2.179017
32	6	0	-4.996724	-5.597618	-4.987049
33	8	0	-6.505654	-5.9394	-6.672532
34	8	0	-2.471525	-5.500407	-5.457599
35	8	0	12.636471	-1.336796	4.897962
36	6	0	12.307734	-1.442483	7.572193
37	1	0	-11.679146	-2.791471	-2.220948
38	1	0	-9.491849	-2.572892	-4.695008

39	1	0	-8.89947	-5.635028	0.165737
40	1	0	-9.377411	-6.864023	-2.905009
41	1	0	-8.851765	-0.730056	0.76329
42	1	0	-9.232158	1.189249	-1.929121
43	1	0	-0.668005	-2.550552	-2.341392
44	1	0	-0.818082	-4.985191	-0.109664
45	1	0	1.322579	-1.280531	1.771157
46	1	0	-1.550067	-1.91006	3.343545
47	1	0	-0.379392	2.175227	-2.939534
48	1	0	2.000378	2.473029	-0.601565
49	1	0	0.561624	6.873598	-1.545339
50	1	0	-3.460827	5.510944	-3.58232
51	1	0	-4.003741	7.915434	-1.348812
52	1	0	-7.148031	4.292685	-1.102668
53	1	0	-5.702046	5.151802	1.718639
54	1	0	-2.595668	3.066313	4.740117
55	1	0	0.644275	2.457923	4.289218
56	1	0	2.023204	8.977173	2.530136
57	1	0	1.8381	7.133847	5.358782
58	1	0	-5.922268	-1.027834	-5.619712
59	1	0	-5.40158	2.114338	-4.712526
60	1	0	-2.864772	-0.063807	-4.760654
61	1	0	-5.770635	-9.514868	-1.796578
62	1	0	-2.684057	-8.284552	-1.401645
63	1	0	-4.968312	-7.896991	1.028697
64	1	0	8.582157	1.526326	6.563573
65	1	0	10.29135	1.57345	-2.658839
66	1	0	6.711103	4.07651	-0.881766
67	1	0	14.232796	-2.027777	1.329411
68	1	0	-2.291009	-5.724966	-7.269265
69	1	0	12.500873	0.441124	8.417349
70	1	0	10.455331	-2.236321	8.060324
71	1	0	13.799457	-2.676998	8.279099
